# Patient-derived monoclonal antibody neutralizes SARS-CoV-2 Omicron variants and confers full protection in monkeys

**DOI:** 10.1038/s41564-022-01198-6

**Published:** 2022-07-25

**Authors:** Craig Fenwick, Priscilla Turelli, Dongchun Ni, Laurent Perez, Kelvin Lau, Cécile Herate, Romain Marlin, Erica Lana, Céline Pellaton, Charlène Raclot, Line Esteves-Leuenberger, Jérémy Campos, Alex Farina, Flurin Fiscalini, Nathalie Dereuddre-Bosquet, Francis Relouzat, Rana Abdelnabi, Caroline S. Foo, Johan Neyts, Pieter Leyssen, Yves Lévy, Florence Pojer, Henning Stahlberg, Roger LeGrand, Didier Trono, Giuseppe Pantaleo

**Affiliations:** 1 Service of Immunology and Allergy, Department of Medicine, Lausanne University Hospital and University of Lausanne, Lausanne, Switzerland; 2 School of Life Sciences, Ecole Polytechnique Fédérale de Lausanne, Lausanne, Switzerland; 3 School of Basic Sciences, Ecole Polytechnique Fédérale de Lausanne and Faculty of Biology and Medicine, UNIL, Lausanne, Switzerland; 4 CEA, Université Paris Sud 11, INSERM U1184, Center for Immunology of Viral Infections and Autoimmune Diseases, IDMIT Department, IBFJ, Fontenay-aux-Roses, France; 5 KU Leuven Department of Microbiology, Immunology and Transplantation, Rega Institute for Medical Research, Laboratory of Virology and Chemotherapy, Leuven, Belgium; 6 VRI, Université Paris-Est Créteil, Faculté de Médicine, INSERM U955, Créteil, France; 7 Inserm U955, Equipe 16, Créteil, France; 8 AP-HP, Hôpital Henri-Mondor Albert-Chenevier, Service d’Immunologie Clinique et Maladies Infectieuses, Créteil, France; 9 Swiss Vaccine Research Institute, Lausanne University Hospital and University of Lausanne, Lausanne, Switzerland

**Keywords:** Viral infection, Antibody therapy

## Abstract

The SARS-CoV-2 Omicron variant has very high levels of transmission, is resistant to neutralization by authorized therapeutic human monoclonal antibodies (mAb) and is less sensitive to vaccine-mediated immunity. To provide additional therapies against Omicron, we isolated a mAb named P2G3 from a previously infected vaccinated donor and showed that it has picomolar-range neutralizing activity against Omicron BA.1, BA.1.1, BA.2 and all other variants tested. We solved the structure of P2G3 Fab in complex with the Omicron spike using cryo-electron microscopy at 3.04 Å resolution to identify the P2G3 epitope as a Class 3 mAb that is different from mAb-binding spike epitopes reported previously. Using a SARS-CoV-2 Omicron monkey challenge model, we show that P2G3 alone, or in combination with P5C3 (a broadly active Class 1 mAb previously identified), confers complete prophylactic or therapeutic protection. Although we could select for SARS-CoV-2 mutants escaping neutralization by P2G3 or by P5C3 in vitro, they had low infectivity and ‘escape’ mutations are extremely rare in public sequence databases. We conclude that this combination of mAbs has potential as an anti-Omicron drug.

## Abstract (web summary)

A potent mAb shows promise in monkeys either alone or in a combination therapy for either prophylaxis or treatment of infection with SARS-CoV-2 Omicron BA.1, BA.1.1 and BA.2.

## Main

SARS-CoV-2 is responsible for >340 million confirmed infections and >5.5 million fatalities worldwide^1^. Its propagation has resulted in the emergence of variants of concern (VOC) that are more transmissible and are resistant to immune responses. VOCs harbouring a high number of mutations compared with the original SARS-CoV-2 strain predominate, with Delta (B.1.617.2) and its 11 to 15 spike mutations now largely replaced by the highly infectious Omicron variant (B.1.1.529.1), which contains up to 37 amino acid mutations in spike protein^2,3^. Fifteen of the spike substitutions in Omicron are in the receptor binding domain (RBD), the region targeted by neutralizing antibodies (whether induced by infection or current vaccines) that were all raised against the original 2019-nCoV Wuhan strain^4–8^. Omicron also resists neutralization by most anti-SARS-CoV-2 mAbs reported so far^9–15^ and is now circulating as several sub-variants including BA.1.1, BA.2 (B.1.1.529.2) and BA.3 (B.1.1.529.3)^2^, creating an urgent unmet medical need for both prophylaxis and therapeutics.

## Results

### P2G3 is a potent monoclonal neutralizing antibody

**Extended Data Fig. 1:**
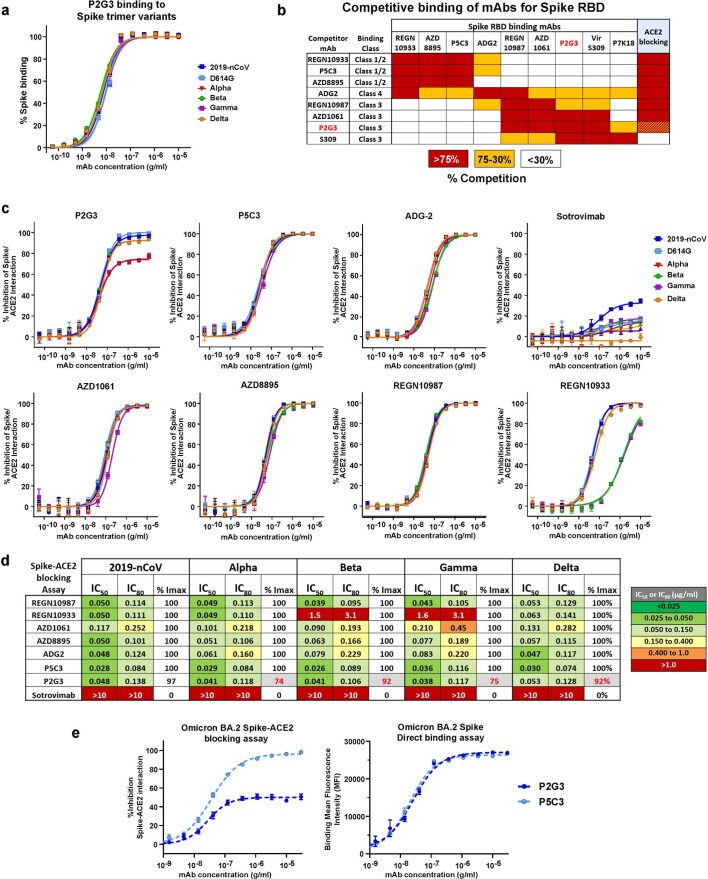
Binding properties of P2G3 and other anti-SARS-CoV-2 antibodies for recombinant Spike trimer proteins from 2019-nCoV and variants of concern in direct binding and Spike−ACE2 interaction assays. **a**) Spike binding curves (n = 2) and **b**) Competitive binding studies between antibodies binding to the 2019-nCoV Spike RBD protein. RBD coupled beads pre-incubated with saturating concentrations of competitor antibody were used for binding studies with mAbs or ACE2. Competitors induced either strong blocking (Red boxes), partial competition (orange boxes) or non-competitive (white boxes) binding with the corresponding mAb to RBD. Red and yellow-hashed lines indicate incomplete blocking of the Spike-ACE2 interaction with Alpha, Gamma and Omicron Spike variant proteins (n = 2 for each mAb curve and Spike proteins). **c**) Spike-ACE2 blocking activity of a panel of in-house, authorized and clinically advanced anti-Spike mAbs and **d**) heatmap showing IC50, IC80 and Imax values for our panel of mAbs in the Spike-ACE2 assay. These Luminex based assays were performed with beads coupled with Spike trimer proteins from the original 2019-nCoV, D614G mutant, Alpha, Beta, Gamma, and Omicron SARS-CoV-2 variants of concern. Sotrovimab was included as a control mAb that binds the RBD without blocking the Spike-ACE2 interaction. **e**) Representative data for Spike-ACE2 blocking activity and Spike binding for P2G3 and P5C3 mAbs using the same Omicron BA.2 Spike trimer coated Luminex beads. Replicates shown for P2G3 and P5C3 are n = 8 and 7 in Spike-ACE2 assay and n = 4 and 2 in the Spike binding assay, respectively. Mean values ± SEM are shown. Source data

We screened for the presence of anti-spike antibodies in serum samples from a cohort of >100 donors and focused on one post-infected donor who received two doses of the mRNA-1273 vaccine and had among the highest serum antibody levels, with excellent breadth against a panel of SARS-CoV-2 variants in a trimeric spike-ACE2 surrogate neutralization assay^16^. Screening of B-cell clone supernatants for high-affinity spike binding led us to prioritize six clones for mAb production via expression of paired heavy and light chains in ExpiCHO cells. During initial profiling of these purified mAbs, P2G3 exhibited the strongest binding affinity for the original 2019-nCoV spike trimer and a panel of spike proteins encoding mutations found in Alpha, Beta, Gamma and Delta VOCs (IC_50s_ of 0.006–0.010 µg ml^−1^) (Extended Data Fig. 1a). Cross-competitive spike RBD binding studies performed with a panel of authorized or clinically advanced anti-SARS-CoV-2 mAbs (REGN10933 and REGN10987 from Regeneron^17^, AZD8895 and AZD1061 from AstraZeneca^18^, ADG-2 from Adagio^19^, S309/Sotrovimab from Vir/GSK^20^) and mAbs previously described by our group^21^ demonstrate that P2G3 binds a unique albeit overlapping epitope with those recognized by both AZD1061 and S309/Sotrovimab, the latter acting by a mechanism distinct from blocking the RBD/ACE2 interaction^20^ (Extended Data Fig. 1b**)**. Importantly, our potent and broadly active Class 1 mAb, P5C3, bound RBD non-competitively with P2G3, prompting us to profile these mAbs both alone and in combination for subsequent studies.

**Fig. 1:**
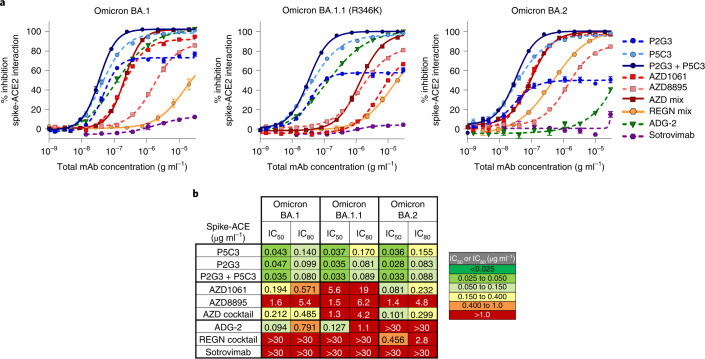
P2G3 is a human mAb with potent activity. **a**, Blocking activity of individual and combinations of anti-spike mAbs in a biochemical spike-ACE2 surrogate neutralization assay using Omicron BA.1, BA.1.1 encoding the R346K mutation and BA.2 variant spike trimer proteins. Mean ± s.e.m. are shown. **b**, Comparative spike-ACE2 blocking activity of P2G3, P5C3 and the P2G3/P5C3 mix compared to a panel of benchmark anti-SARS-CoV-2 mAbs. P2G3 and P5C3 used in these studies and throughout the manuscript contain the LS mutation in the Fc domain (M428L/N434S), previously demonstrated to confer an extended half-life in vivo. Data presented are biological replicates with *n* = 8, 7, 8, 8, 7, 5, 6, 7 and 4 for mAbs in order of appearance in the legend. Source data

Using a biochemical trimeric spike-ACE2 surrogate neutralization assay^16^, we further determined that P2G3 and P5C3 had the most potent and broad activity in blocking ACE2 binding to high quality structural grade spike trimers from all past VOCs compared with our panel of benchmark mAbs (Extended Data Fig. 1c,d). P2G3 and P5C3 also displayed the most potent activity in assays performed with the spike protein from Omicron BA.1, BA.1.1 that includes the R346K mutation and BA.2. (Fig. 1a,b). P2G3 alone gave activities comparable with the P2G3/P5C3 combination, with the cocktail showing 6.1-, 47.2- and 3.4-fold improved IC_80_ values versus the AZD1061/AZD8895 mix, 9.9-, 12.4- and 340-fold improved IC_80_ values versus ADG-2, and 375-, 337- and 32-fold improved IC_80_ values versus the REGN10933/ REGN10987 mix in assays performed with Omicron BA.1, BA.1.1 and BA.2, respectively. Interestingly, while the P2G3/P5C3 combination reached ~100% inhibition in blocking ACE2 binding to Omicron spike proteins at 1 µg ml^−1^ total mAbs, P2G3 alone only blocked 50–72% of ACE2 binding at 20 µg ml^−1^ and P5C3 alone reached a ~100% inhibition at 20 µg ml^−1^.

### Comparison of P2G3 with existing mAb therapies in vitro

**Fig. 2:**
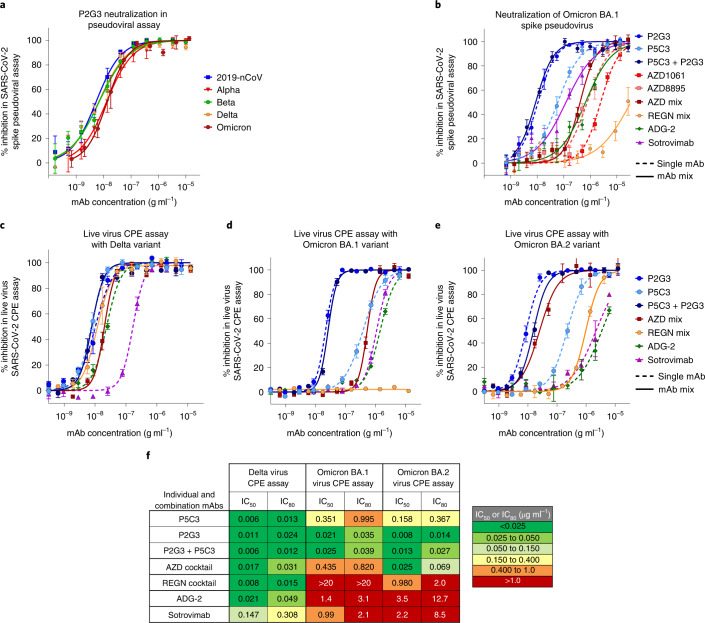
P2G3 neutralizes spike-coated pseudoviruses and live virus VOCs. **a**, Neutralization of lentiviral particles pseudotyped with SARS-CoV-2-spike-expressing VOCs in a 293T-ACE2 infection assay. All spike proteins contained the D614G substitution that became dominant early in the pandemic. Replicates in the concentration response curves were *n* = 5, 5, 6, 8 and 6 for the 2019-nCoV, Alpha, Beta, Delta and Omicron variants, respectively. **b**, Neutralization of lentiviral particles pseudotyped with the Omicron variant spike. Antibody cocktails represent a 1:1 mix of each mAb to give the indicated total mAb concentration. Replicates for mAbs in order of appearance in the legend are *n* = 9, 9, 9, 7, 7, 7, 7, 7 and 6. **c**–**e**, Neutralization activity of P2G3 performed in a live SARS-CoV-2 infectious virus cytopathic effect assay (CPE). The indicated SARS-CoV-2 variants were used to infect Vero E6 cells in vitro in the absence and presence of concentration response of the indicated mAb. Viral inhibition curves are shown for the Delta (**c**, *n* = 4 replicates), Omicron BA.1 (**d**, *n* = 4 replicates) and Omicron BA.2 (**e**, *n* = 6 replicates for P2G3 and P5C3, *n* = 4 for P2G3+P5C3 and *n* = 2 for the remaining mAbs) variants for individual and mAb combinations. **f**, Heat map showing IC_50_ and IC_80_ neutralization potencies for the indicated mAbs in the live virus CPE assays calculated from **c**–**e**. For **a**–**e**, mean ± s.e.m. are shown. Source data

**Extended Data Fig. 2:**
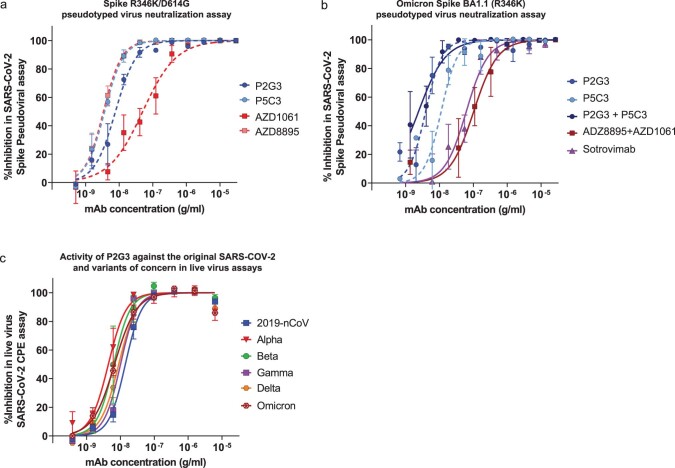
P2G3 retains neutralizing activity against R346K Spike-coated pseudoviruses and all SARS-CoV-2 variants in a live virus cytopathic effect assay. Neutralization of lentiviral particles pseudotyped with: **a**) SARS-CoV-2 Spike encoding R346K mutation and the D614G substitution that became dominant early in the pandemic and **b**) Omicron BA1.1 with the R346K substitution in a 293T-ACE2 infection assay. Panels a and b represent triplicates for all mAbs tested with the exception of AZD8895 and AZD1061 that were tested as duplicates in a. **c**) P2G3 evaluated in live virus cytopathic effect neutralization assays with the original 2019-nCoV and Alpha, Beta, Gamma, Delta and Omicron BA.1 variants of concern with n = 6, 4, 4, 4, 6 and 6 replicates shown, respectively. Mean values ± SEM are shown. Source data

We compared P2G3 alone, or in combination with P5C3, with our panel of current clinically authorized and/or clinically advanced mAbs in pseudovirus neutralization assays. P2G3 had potent neutralizing activity against lentiviruses pseudotyped with spike from the initial 2019-nCoV (D614G), Alpha, Beta and Delta VOCs (IC_80_ value of 0.022, 0.051, 0.038 and 0.035 µg ml^−1^, respectively). Importantly, P2G3 strongly neutralized the Omicron BA.1 spike pseudovirus with an IC_80_ value of 0.038 µg ml^−1^ (Fig. 2a) and thus showed no loss of activity as compared with the other VOCs. In side-by-side comparisons, P2G3 was >42-fold more potent than ADG-2, AZD1061, AZD8895, REGN10933 and REGN10987 mAbs and 19-fold more potent than Sotrovimab at neutralizing Omicron BA.1 spike-pseudotyped lentiviral particles (Fig. 2b). Second most potent was P5C3, with an IC_80_ value of 0.223 µg ml^−1^, and the P2G3/P5C3 combination revealed a minor-enhanced activity over P2G3 alone in this assay, with an IC_80_ value of 0.024 µg ml^−1^ for the total concentration of the two mAbs. Furthermore, P2G3 and P5C3 maintained full neutralizing activity against the ancestral D614G and Omicron BA.1.1 encoding the R346K spike pseudovirus (Extended Data Fig. 2a,b), a mutation present in ~10% of Omicron variant sequences in the GISAID^11^.

### P2G3 neutralization in live virus cytopathic effect assays

We next profiled P2G3 using the initial D614G strain and all current VOCs in a live virus cytopathic effect assay. P2G3 demonstrated broad and potent neutralizing activity with IC_80_ values of 0.028, 0.010, 0.017, 0.021, 0.024 and 0.035 µg ml^−1^ against the 2019-nCoV (D614G) strain, Alpha, Beta, Gamma, Delta and Omicron variants, respectively (IC_80_ ranging from 67–233 pM) (Extended Data Fig. 2c). We went on to compare P2G3 with other mAbs alone or in combinations for their ability to block the Delta and currently prevalent Omicron BA.1 and BA.2 SARS-CoV-2 variants (Fig. 2c–f). All tested mAbs displayed good activity against the Delta variant, although Sotrovimab was ~6× less potent than ADG-2 and >10× less potent than P2G3, P5C3 and both the AZD and REGN cocktails against this virus.

Of note, P2G3 was by far the most active mAb against Omicron BA.1 with an IC_80_ value of 0.035 µg ml^−1^, which is ~23-fold, 60-fold and 88-fold more potent than those of AZD1061/AZD8895, Sotrovimab and ADG-2, respectively, the REGN combination being completely ineffective against this variant (Fig. 2d,f). Although P5C3 had an IC_80_ against Omicron in the range of that of the AZD cocktail, the P2G3/P5C3 combination showed high neutralization activity comparable to P2G3 alone, with an IC_80_ of 0.039 µg ml^−1^ total mAb. P2G3 was again the most potent mAb in neutralizing the Omicron BA.2 variant with an IC_80_ value of 0.014 µg ml^−1^, which is 4.9-fold, 14-fold, 907-fold and 607-fold more potent than AZD1061/AZD8895, REGN10933/REGN10987, ADG-2 and Sotrovimab, respectively (Fig. 2e–f). Importantly, although the spike-ACE2 surrogate neutralization assay correlates well with cell-based neutralization assays^16^, P2G3 only blocked ~50–72% of ACE2 binding to Omicron spike proteins (Fig. 1a) despite P2G3 reaching 100% of the maximum signal in a direct binding assay (Extended Data Fig. 1e). This suggests that at least with the Omicron variant, virus inhibition by P2G3 may not be solely mediated through the spike-ACE2 interaction, but rather through a mechanism analogous to that of S309/Sotrovimab, reported to be via induction of spike trimer cross-linking, steric hindrance, aggregation of virions^20^ and/or inhibition of viral membrane attachment via C-type lectin receptors^22^.

### Antibody-dependent cellular cytotoxicity and antibody-dependent cellular phagocytosis with P2G3

**Extended Data Fig. 3:**
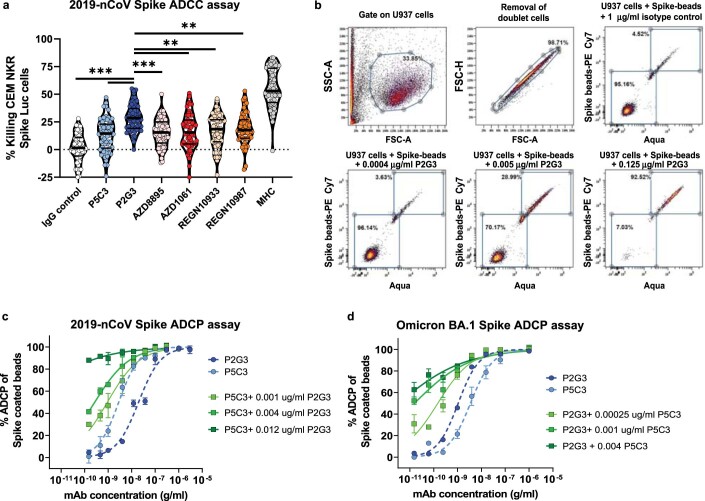
P2G3 LS shows strong Fc-mediated functional activity in ADCC cell killing and ADCP assays. **a**) Antibody dependent cellular cytotoxicity assay (ADCC) performed with CEM NKR Luciferase cells stably expressing cell surface 2019-nCoV Spike. P2G3 exhibits potent ADCC activity in killing Spike positive cells. ADCC experiments performed with five replicates per condition using effector cells from five different healthy donors with each donor evaluated in one to three different experiments (n = 50 tests per condition). CEM NKR Spike cells were incubated with 0.30 µg/ml of the indicated human IgG mAbs. Violin plots are shown with median values as thick middle line and quariles for upper and lower lines. Statistical difference evaluated by two-way ANOVA with p-values presented as p = 0.0096 (**), p = 0.0001 and 0.0002 (from left to right ***) and p < 0.0001 (****). **b**) Flow cytometry gating strategy for the selection of U937 cells, the removal of cell doublets and the evaluation of cells for which Spike-coated fluorescent beads have undergone phagocytosis. The threshold gate for Spike-specific phagocytosis of beads was set using and isotope control antibody and representative dot plots for P2G3 mediated ADCP of 2019-nCoV Spike-coated beads by U937 are shown with >4000 cells analysed per condition. **c**, **d**) Antibody dependent cellular phagocytosis assay performed ancestral 2019-nCoV and with Omicron BA.1 variant Spike protein biotinylated and bound to streptavidin coated fluorescent beads. Beads mixed with the indicated antibody concentrations were incubated with the U937 monocyte effector cell line and antibody dependent cellular phagocytosis of the Spike coated beads was evaluated by flow cytometry. Dashed lines correspond to individual antibodies and solid lines indicate combinations of P2G3 and P5C3. Results shown are representative data for three separate experiments with each concentration response tested in duplicates or triplicates. Mean values ± SEM are shown. Source data

Given the potential importance of antibody functional activity that is mediated through the constant immunoglobulin Fc region and can lead to more efficient virus control and clearance^23–25^, we investigated P2G3 activities in antibody-dependent cellular cytotoxicity (ADCC) and antibody-dependent cellular phagocytosis (ADCP) assays. ADCC enables the targeted killing of cells that display SARS-CoV-2 spike protein at the membrane surface and our in vitro assay uses CEM-NKR cells stably expressing 2019-nCoV spike cultured with primary effector cells from healthy donors in the presence and absence of anti-spike mAbs. P2G3 mAb exhibited a robust ADCC activity that was superior in killing spike-positive cells compared with all other anti-spike mAbs tested (Extended Data Fig. 3a). Although these studies were not performed with an Omicron spike stable cell line, it is generally accepted that ADCC functional activity is maintained against the different SARS-CoV-2 VOCs for mAbs that conserve spike variant binding^26^. We next evaluated ADCP activity using 2019-nCoV or Omicron BA.1 spike trimer-coated fluorescent beads mixed with different concentrations of P2G3 and/or P5C3 then incubated with U937 effector cells. This monocyte cell line expresses high levels of Fc-gamma receptors capable of inducing phagocytosis of opsonized viruses or beads coated with the spike antigen as in our assay (Extended Data Fig. 3b). Used alone, P2G3 and P5C3 mAbs showed ADCP IC_80_ activities of 0.074 and 0.010 µg ml^−1^, respectively, with P5C3 showing ~7-fold greater potency. Conversely, using Omicron spike-coated beads, P2G3 exhibited potent ADCP activity that was 3-fold improved relative to that of P5C3. In studies performed with both ancestral and Omicron spike, the P2G3/P5C3 mix shows enhanced ADCP activities compared with mAbs used individually (Extended Data Fig. 3c,d). Of note, P2G3 and P5C3 used in these Fc-mediated functional activity assays and throughout the Article contain the LS mutation in the Fc domain (M428L/N434S), previously demonstrated to confer an extended half-life in vivo^27^, a highly desirable feature for prophylactic use.

### Prophylactic use of P2G3 in hamster infection model

**Extended Data Fig. 4:**
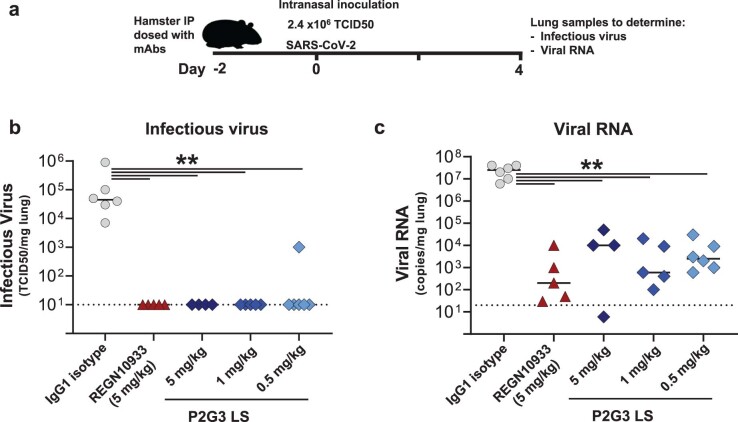
P2G3 LS confers potent in vivo efficacy in the hamster challenge model for SARS-CoV-2 infection. **a**) Overview of study design for the SARS-CoV-2 hamster challenge model. Animals were administered intraperitoneally 5.0, 1.0 or 0.5 mgkg−1 of P2G3, 5 mg/kg of REGN10933 positive control or 5 mg/kg of an IgG1 isotype control and challenged two days later (Day 0) with an intranasal inoculation of the original 2019-nCoV SARS-CoV-2 virus (2.4 ×106 TCID50). **b**) Median levels of infectious virus and **c**) viral RNA copies/mg lung tissue in each of the study arms are shown on day 4 post-inoculation with SARS-CoV-2 virus. Treatment arms for IgG control, REGN10933 5 mg/kg, P2G3 5 mg/kg, P2G3 1.0 mg/kg and P2G3 0.5 mg/kg consist of n = 6, 5, 4, 5, and 6 hamsters, respectively. Non-parametric Mann-Whitney U- two-sided tests were used to evaluate the statistical difference between the treatment conditions with p = 0.0043, 0.0095, 0.0043 and 0.0022 (from left to right, **) in b and p = 0.0043, 0.0095, 0.0043 and 0.0022 (from left to right, **) in c. Source data

Having demonstrated the superior in vitro neutralizing activity of P2G3, we next evaluated the neutralizing potency of P2G3 in vivo in a prophylactic hamster challenge model of SARS-CoV-2 infection. Antibody dosed animals were challenged 2 d later with an intranasal inoculation of the original 2019-nCoV SARS-CoV-2 virus (Extended Data Fig. 4a) and then 4 d later, hamster lung tissue was monitored for infectious virus and viral RNA. Infectious virus was undetectable in lungs from almost all P2G3-treated hamsters, with only 1 of 6 hamsters in the lowest dosed 0.5 mg kg^−1^ group showing reduced although detectable levels of infectious virus compared with the isotype mAb-treated control animals (Extended Data Fig. 4b). Complete prophylactic protection was observed with P2G3 mAb plasma levels >6.2 µg ml^−1^ at the time of viral inoculation and P2G3 treatment groups showed a significant ~4-log reduction of genomic viral RNA levels (Extended Data Fig. 4c**)**.

### P2G3 therapeutic efficacy in cynomolgus macaques

**Fig. 3:**
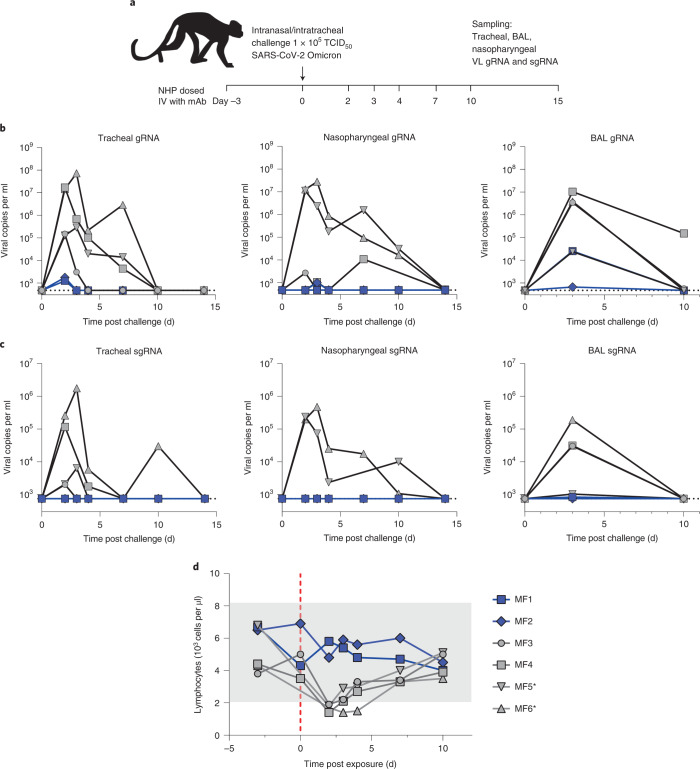
In vivo efficacy in the NHP challenge model. **a**, Overview of study design for the SARS-CoV-2 NHP challenge model. Animals MF1 and MF2 were intravenously administered 10 mg kg^−1^ of P2G3 and challenged 3 d later (day 0) along with control animals MF3 and MF4 via intranasal and intratracheal inoculation of the Omicron BA.1 SARS-CoV-2 virus (1 × 10^5^ TCID_50_). **b**,**c**, Tracheal swabs (left), nasopharyngeal swabs (middle) and bronchoalveolar lavages (BAL, right) performed during the course of the study were evaluated for viral copies per ml of gRNA (**b**) and sgRNA (**c**), with data plotted to include two historical control animals (MF5* and MF6*) infected with the same inoculum of Omicron virus. **d**, Flow cytometry analysis of blood samples from NHPs collected throughout the study shows strong lymphopenia in control animals following challenge with Omicron SARS-CoV-2, while P2G3 LS-treated monkeys show stable levels of lymphocytes. Dotted line indicates lower limit of detection at 2.68- and 2.87-log copies per ml for viral gRNA and sgRNA, respectively. Source data

We next evaluated P2G3-mediated protection from SARS-CoV-2 Omicron BA.1 infection in a cynomolgus macaque pre-exposure challenge study. Monkeys (*n* = 2) were administered 10 mg kg^−1^ of P2G3 LS intravenously and challenged 72 h later via combined intranasal and intratracheal routes with 1 × 10^5^ 50% tissue culture infectious dose (TCID_50_) of SARS-CoV-2 B.1.1.519 Omicron BA.1 virus (Fig. 3a). Following viral challenge, control animals (*n* = 4) showed similar genomic (g)RNA levels and kinetics, with median peak viral loads (VL) of 6.9- and 6.6-log_10_ copies per ml gRNA at 2–3 d post challenge in tracheal swabs and bronchoalveolar lavage (BAL) samples, respectively (Fig. 3b). Nasopharyngeal swabs showed a higher-level variability in VL between control animals but still showed median peaks of 6.9-log_10_ copies per ml for gRNA. In comparison, the two P2G3 LS-treated monkeys had a strong median peak ΔVL reduction of 3.8-, 2.5- and 3.9-log_10_ copies per ml gRNA for tracheal, nasopharyngeal and BAL samples, respectively.

Active viral replication, as assessed by subgenomic (sg)RNA levels, peaked at 2–3 d post challenge, with tracheal swabs and BAL showing median values of 4.8- and 4.5-log_10_ copies per ml, respectively, and nasopharyngeal samples showing variable responses ranging from undetectable (<2.9-log_10_) to 5.4-log_10_ copies per ml (Fig. 3c). P2G3 LS-treated monkeys had sgRNA levels that were at or below the limit of detection, exhibiting 1.9-, 2.2- and 1.6-log_10_ reduced levels in tracheal, nasopharyngeal and BAL samples, respectively. Consistent with viral protection resulting in reduced detection of gRNA and sgRNA, P2G3-treated monkeys exhibited stable lymphocyte levels throughout the study, whereas strong lymphopenia, determined by lymphocyte levels below 2.1 × 10^3^ cells per µl, was observed in all control animals challenged with the Omicron variant of SARS-CoV-2 (Fig. 3d).

### P2G3/P5G3 combination has in vivo therapeutic efficacy

**Fig. 4:**
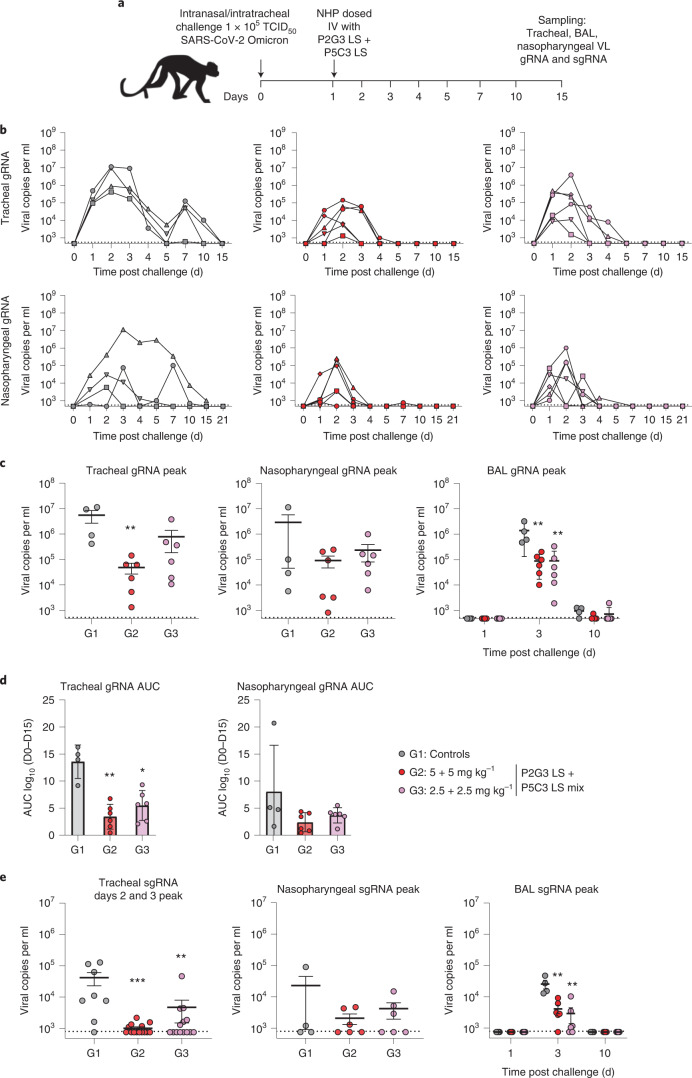
Combination therapy with P2G3 and P5G3 in NHPs. **a**, Overview of study design for the therapeutic SARS-CoV-2 NHP challenge model. Animals were challenged through intranasal and intratracheal inoculation of the Omicron BA.1 SARS-CoV-2 virus (1 × 10^5^ TCID_50_) on day 0 and 24 h later, group 2 (G2, red circles, *n* = 6) NHP were intravenously administered 5 mg kg^−1^ of P2G3 LS and 5 mg kg^−1^ of P2G3 LS, group 3 (G3, pink circles, *n* = 6) were administered 2.5 mg kg^−1^ of P2G3 LS and 2.5 mg kg^−1^ of P2G3 LS, and group 1 (G1, grey circles, *n* = 4) were used as untreated reference controls. **b**–**e**, Tracheal swabs, nasopharyngeal swabs and bronchoalveolar lavages (BAL) performed during the course of the study were evaluated for viral copies per ml of gRNA (**b**–**d**) and sgRNA (**e**). Study groups were evaluated for peak gRNA (**c**), area under the curve for gRNA (**d**) and sgRNA peak viral copies per ml (**e**) in the different samples collected and at different timepoints. Dotted line indicates lower limit of detection at 2.68- and 2.87-log copies per ml for viral gRNA and sgRNA, respectively. Mean ± s.e.m. are shown. Mann-Whitney two-sided tests were performed to compare study groups in **c**, **d** and **e**; **P = 0.0095 for all in **c**; **P = 0.0095 and *P = 0.019 for **d**; ***P = 0.0005, **P = 0.0062, 0.0095, 0.0095 (from left to right) for **e**. Source data

In a second study to evaluate mAb therapeutic efficacy, monkeys were challenged with SARS-CoV-2 B.1.1.519 Omicron BA.1 virus as above and then 24 h post challenge, animals in three groups were either left untreated (*n* = 4) or administered a P2G3 LS + P5C3 LS combination at 5 + 5 mg kg^−1^ (*n* = 6) or 2.5 + 2.5 mg kg^−1^ (*n* = 6) through a single intravenous injection (Fig. 4a). Tracheal, nasopharyngeal and BAL samples collected longitudinally for each monkey were evaluated for both gRNA (Fig. 4b) and sgRNA (Supplementary Fig. 1) viral copies per ml. Consistent with the prophylactic challenge study, control animals showed comparable gRNA levels and kinetic profiles, with median peak VL of 6.7- and 6.1-log_10_ copies per ml at 2–3 d post challenge in tracheal swabs and BAL samples, and elevated but more variable nasopharyngeal VL levels of 6.5-log_10_ copies per ml gRNA (Fig. 4c). Importantly, monkeys in the 5 + 5 mg kg^−1^ P2G3 LS + P5C3 LS combination treatment arm showed strong, significantly reduced median peak ΔVL of 2.1- and 1.5-log_10_ for tracheal and BAL samples (*P* = 0.0095 for each), respectively, and 1.2-log_10_ copies per ml gRNA for nasopharyngeal samples (Fig. 4c**)**. Similarly, monkeys treated with the 2.5 + 2.5 mg kg^−1^ lower dose mAb combination showed median peak ΔVL reduction of 0.85- 1.1- and 1.2-log_10_ copies per ml gRNA for tracheal, BAL and nasopharyngeal samples, respectively, with BAL samples showing significantly reduced levels (*P* = 0.0095) (Fig. 4c). Therapeutic efficacy was furthermore demonstrated by monitoring area under the curve (AUC) levels of gRNA, where the high and low dosed P2G3 LS + P5C3 LS combination exerted significant 4- and 2.5-fold reduction in virus detected in the trachea (*P* = 0.0095 and 0.0190, respectively) and 2.5- and 2.2-fold reduced gRNA AUC levels, respectively, in nasopharyngeal fluids relative to the untreated control monkeys (Fig. 4d**)**.

Active viral replication was also significantly inhibited in the high and low dose treatment arms, with tracheal swabs showing 41- and 8.8-fold reduction in sgRNA on days 2/3 post challenge (*P* = 0.0005 and 0.0062), respectively (Fig. 4e). P2G3 LS + P5C3 LS treatment combinations furthermore reduced sgRNA in BAL samples by 6.2- and 8.8-fold compared with the untreated control monkeys (*P* = 0.0095 for both) (Fig. 4e**)**. Overall, these studies strongly support the therapeutic efficacy of the P2G3 LS + P5C3 LS combination in clearing virus and inhibiting active viral replication of the highly relevant Omicron BA.1 variant.

### Cryo-electron microscopy structure of P2G3 and P5C3 Fab bound to spike trimer

**Fig. 5:**
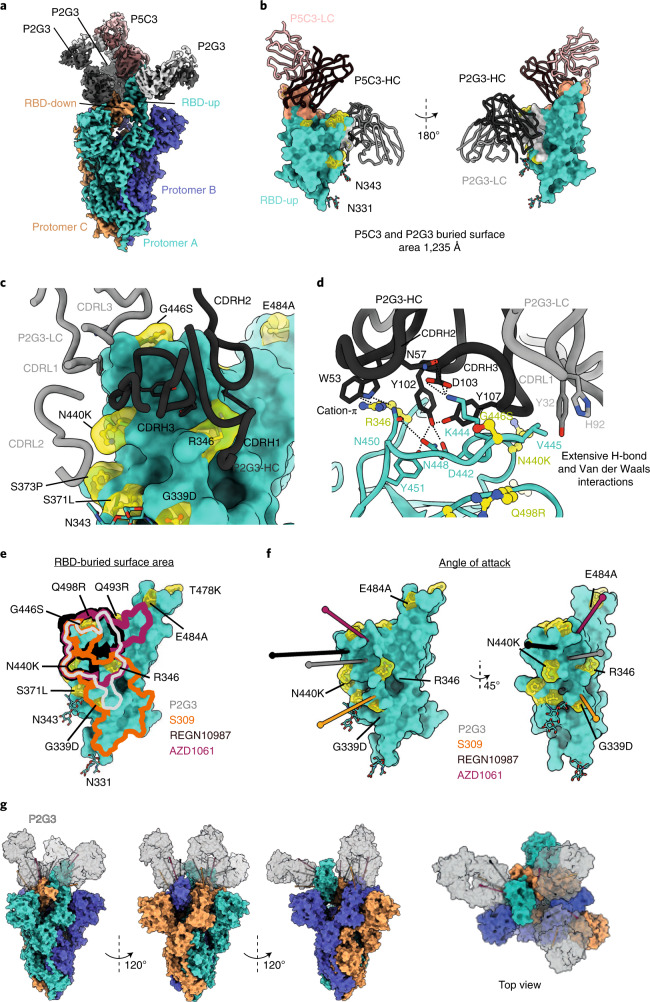
P2G3 and P5C3 bind the full-length Omicron spike. **a**, Cryo-EM composite density map of the full-length Omicron spike bound to one P5C3 and three P2G3 Fab fragments. Spike protomers are coloured in green, orange and blue, P5C3 Fabs in dark and light orange, P2G3 in black and grey. **b**, Surface representation of the RBD in the up configuration (green) bound to both P5C3 and P2G3, with heavy and light chains depicted as liquorice ribbons. The buried surface area formed by the Fabs are depicted on the RBD surface and coloured in grey for P2G3 and orange for P5C3. The Omicron mutations are shown in yellow as balls-and-sticks and transparent surfaces. The N-linked glycans at asparagine 331 and 343 are shown as sticks. The right panel shows a 180° rotation of the RBD/P2G3/P5C3 complex relative to the left panel. **c**, Zoomed-in view of the interacting region of P2G3 with CDR loops of the heavy and light chains specified. Omicron mutations are highlighted in yellow. **d**, Detailed analysis of the interactions between the Omicron RBD shown as ribbons (green) and the P2G3 Fab heavy and light chains shown as liquorice (black and grey). Residues at the interface are shown as sticks, with potential interactions of interest shown as dashed lines. Omicron mutations are shown as balls-and-sticks in yellow. **e**, Structures of several class 3 antibodies (Fabs) bound to RBDs were superimposed on the Omicron RBD. The buried surface area formed by the indicated Fabs are outlined on the RBD surface and coloured correspondingly (Fab-RBD structures AZD1061, PDB-7L7E; REGN10987, PDB-6XDG; S309, PDB-7BEP). The Omicron mutations are shown in yellow as balls-and-sticks and transparent surfaces. **f**, Fab binding angle of attack to the RBD is defined as the line connecting the centroid of the Fab to the centroid of the surface area of the RBD that the Fabs bury. The angle of attack of P2G3 is compared to that of other class 3 antibodies, viewed from multiple angles. RBD is in green, Omicron mutations in yellow. **g**, P2G3 binding to the full Omicron trimer was modelled by superimposing the Fabs onto the RBD of each protomer. The complex is shown from different sides and top view. P2G3 Fabs are able to bind all RBD-up and RBD-down conformations simultaneously.

**Extended Data Fig. 5:**
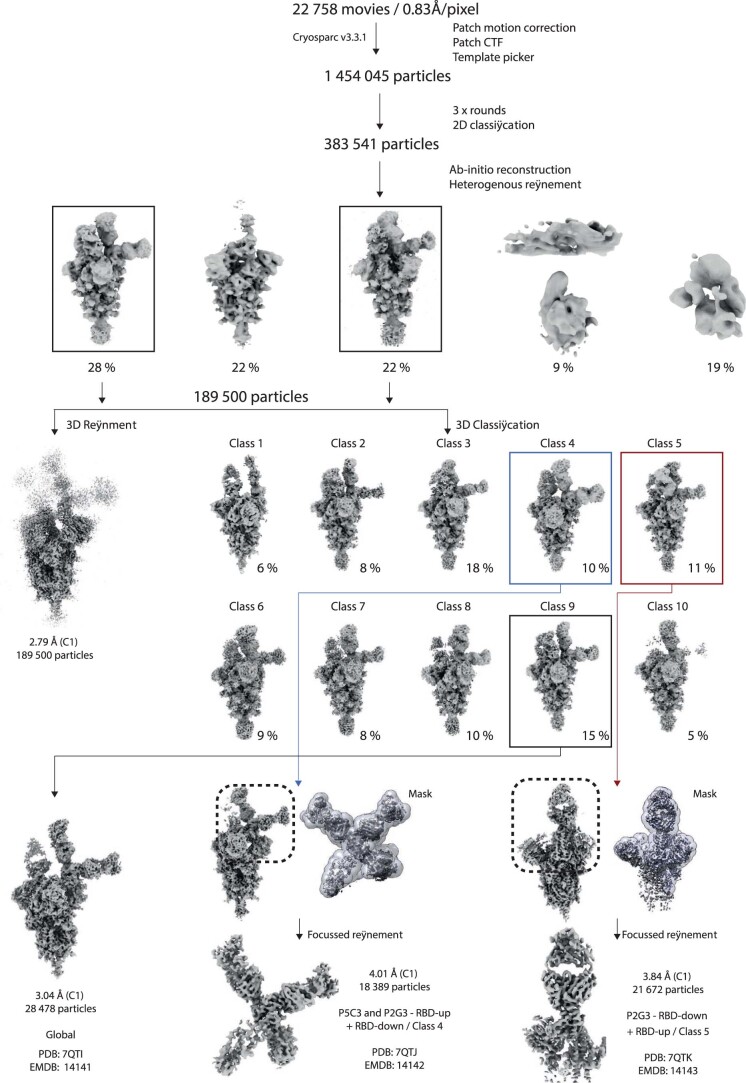
Cryo-EM processing of the Omicron Spike. Cryo-EM processing workflow performed in CryoSPARC v.3.3.1.

**Extended Data Fig. 6:**
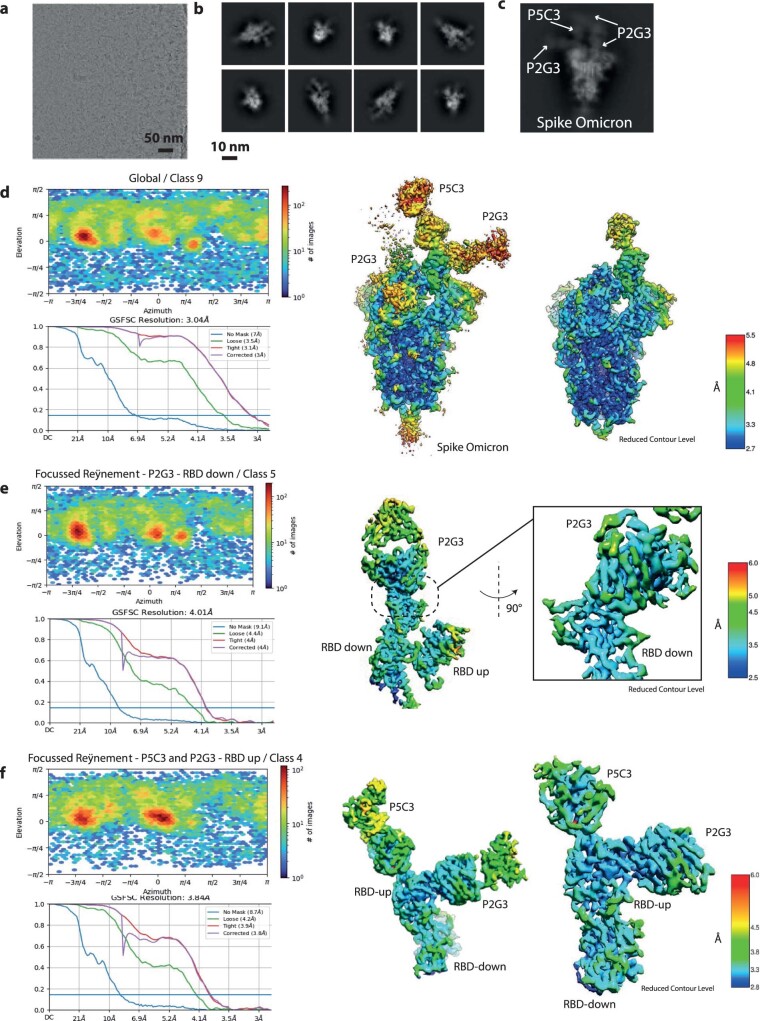
Details of Cryo-EM processing and Resolution of Cryo-EM maps. **a**) Representative micrograph represents one out of a total of 22 758 micrographs (See Table 1) taken from 1 grid. **b**) Representative 2D class averages. **c**) Enlarged 2D class showing the Omicron Spike with bound Fabs. **d**) Direction distribution plot and FSC curves indicating a resolution of 3.04 Å (FSC 0.143) of the Global (Class 9) map off the full-length Omicron Spike bound to Fabs. **e**) Direction distribution plot and FSC curves indicating a resolution of 3.84 Å (FSC 0.143) of the P2G3-bound-RBD-down (Class 5) locally refined map **f**) Direction distribution plot and FSC curves indicating a resolution of 4.01 Å (FSC 0.143) of the P5C3-P2G3-bound-RBD-up (Class 4) locally refined map. Global and focused refined maps coloured by local resolution.

**Extended Data Fig. 7:**
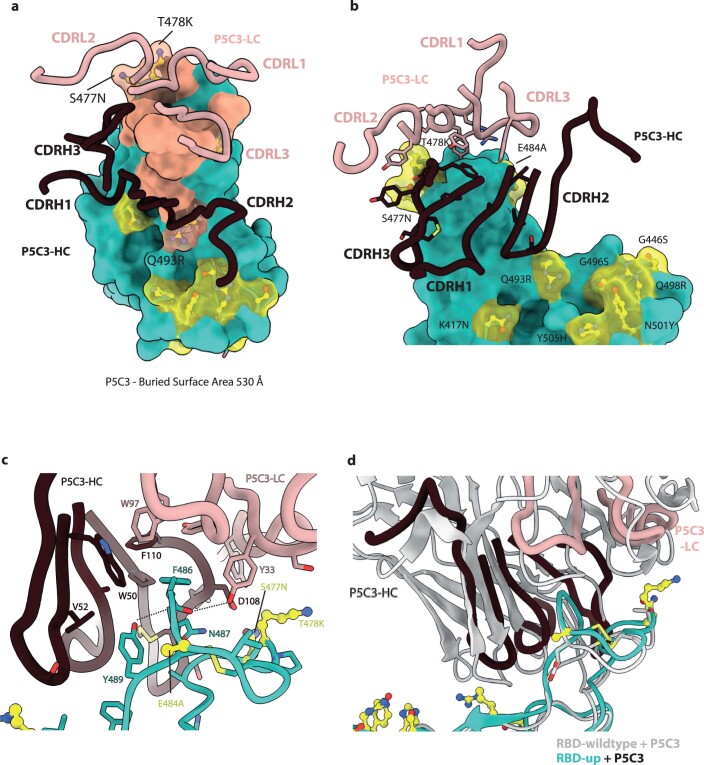
Analysis of the P5C3 Fab-Omicron RBD interacting surfaces. **a**) The buried surface area of P5C3 (pink) overlaid on the RBD surface (green). P5C3 buries around 500 Å of the surface of the Omicron RBD. Specific CDR loops of the heavy and light chains are indicated. Omicron mutations are shown as balls-and-sticks and transparent surfaces in yellow. **b**) Zoomed-in view of the interacting region of P2G3. Specific CDR loops of the heavy and light chains are indicated. Omicron mutations in the region of the Fab are highlighted in yellow. Interacting residues of the Fab are shown as sticks. **c**) Detailed atom level analysis of the interactions between the Omicron RBD shown as ribbons (green) and the P5C3 Fab heavy and light chains shown as liquorice (dark red and pink). Residues at the interface are shown as sticks with potential interactions of interest as dashed lines. Omicron mutations are shown in yellow. **d**) Superposition of the P5C3-Omicron-RBD interface with the P5C3-wild-type-RBD interface (PDB; 7PHG).

**Extended Data Fig. 8:**
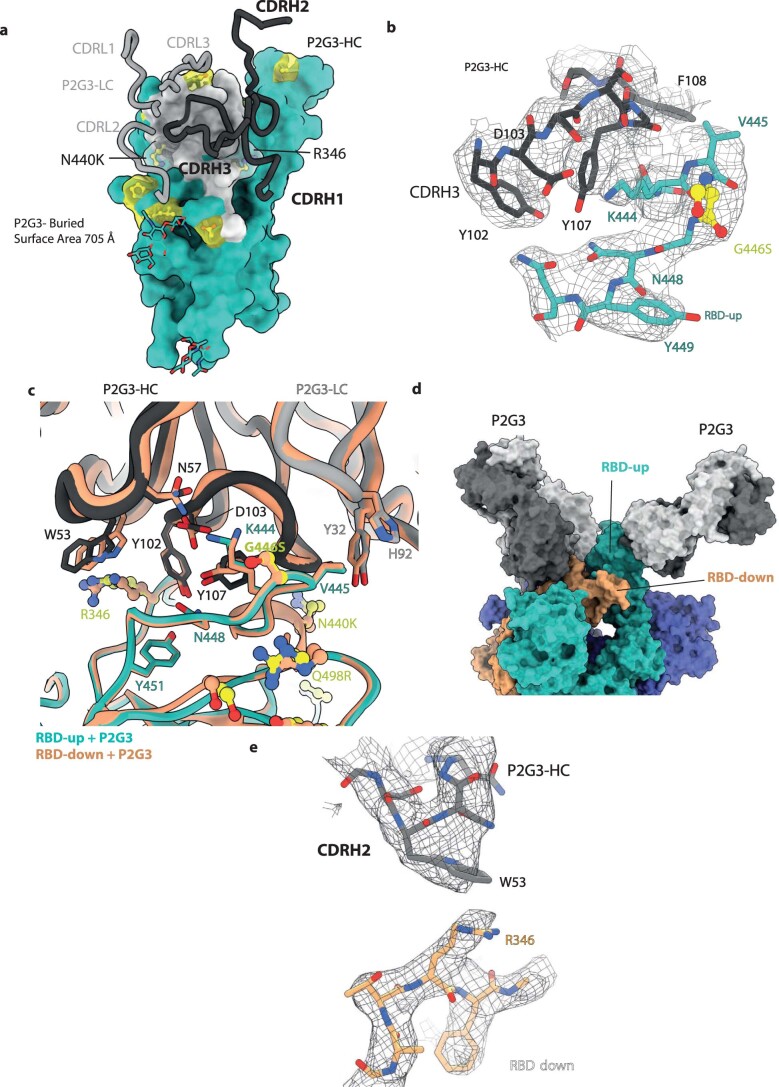
Additional views of the P2G3 Fab-Omicron RBD interacting surfaces. **a**) The buried surface area of P2G3 (grey) overlaid on the RBD surface (green). P2G3 buries 705 Å of surface of the Omicron RBD. Specific CDR loops of the heavy and light chains are indicated. Omicron mutations are shown as balls-and-sticks and transparent surfaces in yellow. **b**) Stick representation of the P2G3 interface of CDRH3 and the RBD region containing residues 440-451. The mesh represents the Cryo-EM density. **c**) Superposition of the P2G3-RBD-up interface with the P2G3-RBD-down interface shows no significant differences with interactions conserved. **d**) Binding of P2G3 Fabs to the RBD-up domain in green and RBD-down domain in orange within the trimeric Spike. **e**) Stick representation of the P2G3 interface of CDRH2 residue W53 that forms a potential cation-pi interaction with RBD residue R346. The mesh represents the Cryo-EM density.

**Extended Data Fig. 9:**
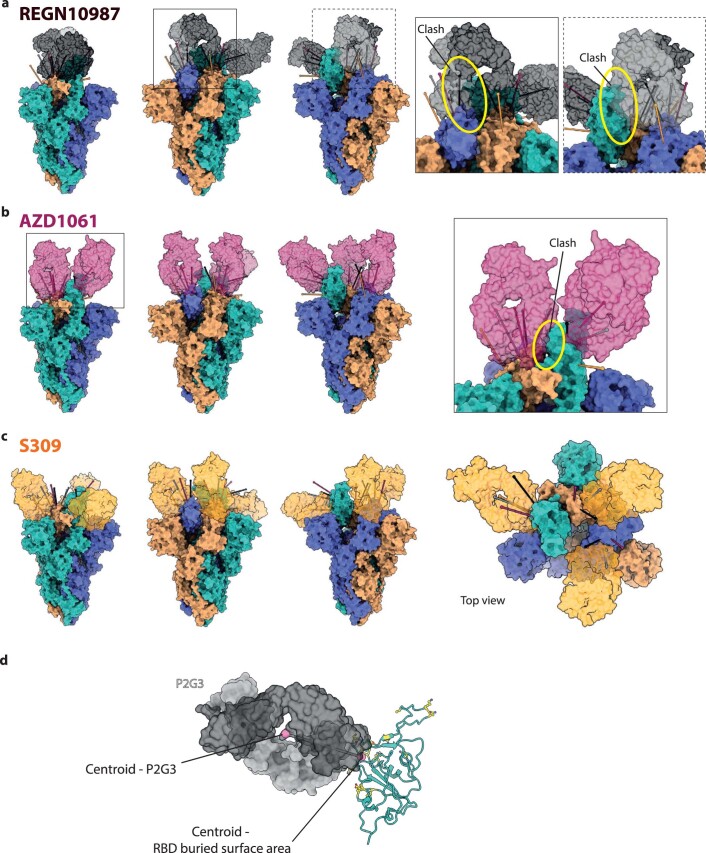
Angle of attack of Fabs for authorized and clinically advanced anti-SARS-CoV-2 mAbs modelled to the Omicron Spike trimer. Model of Fabs for Class 3 antibodies, REGN10987, AZD1061 and S309/Sotrovimab binding each of the RBD protomers for the full Omicron trimer. Trimers are shown from multiple perspectives to visualize the different angles of attack the Fabs have depending on the Spike protomer. **a**) REGN10987 Fab bind to the green RBD-up conformation but modelled REGN10987 Fab bound to either the RBD-down of the orange or blue protomer would clash sterically with the adjacent blue RBD-down or green RBD-up protomers, respectively. Together it is predicted REGN10987 is able to bind only RBD-up. **b**) AZD1061 is predicted to bind the RBD-up form and RBD-down form of the blue protomer but AZD1061 Fab modelled binding to the RBD-down of the orange protomer would potentially clash with the adjacent green RBD-up. **c**) S309/Sotrovimab is able to bind all RBDs simultaneously as shown for P2G3. **d**) Angle of attack of Fabs to the RBD is defined as the line connecting the centroid of the Fab to the centroid of the surface area of the RBD that the Fabs bury.

To decipher the molecular features underlying P2G3 and P5C3 potent neutralization of Omicron spike, we performed single-particle cryo-electron microscopy (cryo-EM) reconstruction of the Omicron spike trimeric ectodomain^12,28,29^ bound to both Fabs, at a 3.04 Å resolution (Fig. 5a, Extended Data Figs. 5 and 6, and Supplementary Data Table 1). We found the Fabs to bind simultaneously at distinct sites on the trimer, with a majority of images revealing three P2G3 Fabs bound to either up- or down-RBD conformations and one P5C3 Fab bound to an up-RBD. The region of the Omicron RBD interacting with the Class 1 P5C3 mAb is identical to that previously described for the D614G spike (Extended Data Figs. 5–7)^21^. P2G3 binds a surface area of around 700 Å^2^ as a Class 3 neutralizing mAb^4^, recognizing an epitope on the SARS-CoV-2 RBD distinct from the receptor-binding motif, with the two mAbs together covering a surface >1,200 Å^2^ (Fig. 5b and Extended Data Figs. 7a and 8a). To characterize the P2G3 paratope and epitope interface in detail, we performed local refinement of the P2G3 Fab-RBD interacting region and reached a resolution of 3.84 Å with well-defined density, allowing clear interpretation of sidechain positions (Extended Data Figs. 6 and 8, and Supplementary Fig. 2 and Data Table 1). The P2G3 paratope is composed of four complementarity-determining region (CDR) loops binding at the back of the RBD. The interactions are mediated through electrostatic and hydrophobic contacts (Fig. 5c,d and Extended Data Fig. 8b,c) and involve 16 residues of the RBD, mainly bound by the heavy chain of the P2G3 mAb. The 18-residue-long CDRH3 sits at the top of a loop that comprises residues 344–347, and also contacts the amino acids at the limits of the 5-stranded β-sheet (residues 440–451), overall accounting for >60% of the buried surface area (431 Å^2^) (Extended Data Fig. 8a–c). The interactions between P2G3 and the Omicron RBD are conserved in both RBD-up and RBD-down states (Extended Data Fig. 8c,d). CDRH2 extends the epitope by interacting with R346 that is engaged by residue W53 via a potential cation-pi interaction (Fig. 5d and Extended Data Fig. 8e), an interaction that is probably conserved with the R346K spike substitution (Extended Data Fig. 2a,b). The only potential contact from the light chain derives from the CDRL1 Y32 forming a hydrophobic interaction with V445 of the RBD (Extended Data Fig. 8c). Moreover, P2G3 is only observed to contact RBD amino acid residues and the distance to the nearest atom of the glycan branch is ~10 Å from P2G3. Importantly, the epitope defined by our structural studies rationalizes the potent neutralizing activity of P2G3 against the Omicron variant relative to other Class 3 mAbs. Omicron mutations S371L, N440K, G446S and the minor R346K sub-variant are all situated outside of, adjacent to or have little effect on recognition of the P2G3-binding epitope, whereas two or more of these mutations directly impinge on epitopes recognized by REGN10987, AZD1061 and S309/Sotrovimab (Fig. 5e). Furthermore, P2G3 displays a unique binding orientation on the RBD, with its Fab angling away from most of these Omicron mutations (Fig. 5f). In modelling the observed angles of attack of various Class 3 mAbs, it is possible that REGN10987 only binds to the up-RBD form while AZD1061 binds one up- and one down-RBD form, with steric hindrance blocking the third RBD site on the Omicron spike trimer (Extended Data Fig. 9). In contrast, P2G3 and S309/Sotrovimab probably binds both the up and down forms of Omicron RBD without clashes (Fig. 5g and Extended Data Fig. 9c), a characteristic that may contribute to the largely conserved and high potency of P2G3 across VOCs.

### Cross-neutralization of P2G3 and P5C3 escape mutants

**Fig. 6:**
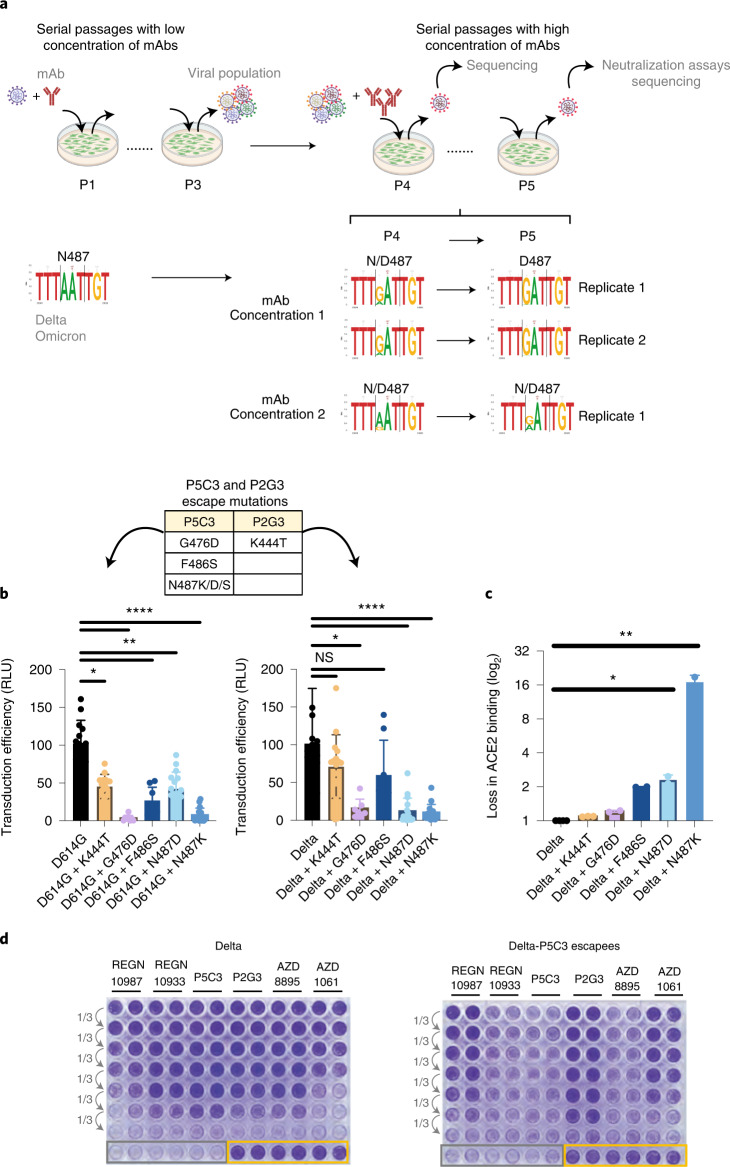
Identification and characterization of escape mutations to P2G3 and P5C3. **a**, Schematic representation of escapees selection. Delta and Omicron replicative isolates were used to infect Vero E6 cells (MOI of 0.2), each in duplicate, in the presence of suboptimal concentrations of antibodies. Supernatants were collected, diluted 40-fold and used to infect cells for two more passages in the same conditions (P1 to P3). Putative viral escapees were further selected by serial passages of 2-fold diluted supernatants pre-incubated with high concentrations of antibodies (three concentrations, each tested in duplicate). Viral RNA extracted from supernatants collected at each passage was deep-sequenced and P5 viral supernatant used for CPE-based neutralization assays. **b**, Mutations identified across escape selection experiments are indicated in the table. Lentivectors pseudotyped with spikes mutated on the identified residues were produced in parallel with stocks adjusted for p24 content and the same amount of each lentivector used to transduce 293T ACE2 cells. In the D614G panel, replicates from left to right were *n* = 36, 36, 8, 8, 28 and 28. The Delta variant panel has *n* = 39, 36, 8, 8, 34 and 34 replicates, respectively. Transduction efficiency was monitored by luciferase activity (RLU) in the transduced cells. **c**, The direct binding of ACE2 to mutated trimeric spikes was monitored by Luminex-based binding assays (*n* = 2–3 biological replicates). **d**, Vero E6 cells were infected in duplicates with normalized amounts of Delta or Delta P5C3 escapees collected from the escapees selection experiments and pre-incubated or not with 3-fold serial dilutions of mAbs as indicated. Cytopathic effect was monitored 2 d later with crystal violet staining of the live cells. Grey and orange squares: cells infected with viruses in the absence of mAbs and non-infected cells, respectively. Kruskal-Wallis tests with Dunn’s multiple-comparison correction was performed to compare wild type and mutants: for **b**, *P = 0.028 and 0.045 (from left to right), **P = 0.0026 and 0.0086 (from left to right) and ****P < 0.0001; NS, not significant; for **c**, *P = 0.046 and **P = 0.0088. Error bars represent mean ± s.d. Source data

**Extended Data Fig. 10:**
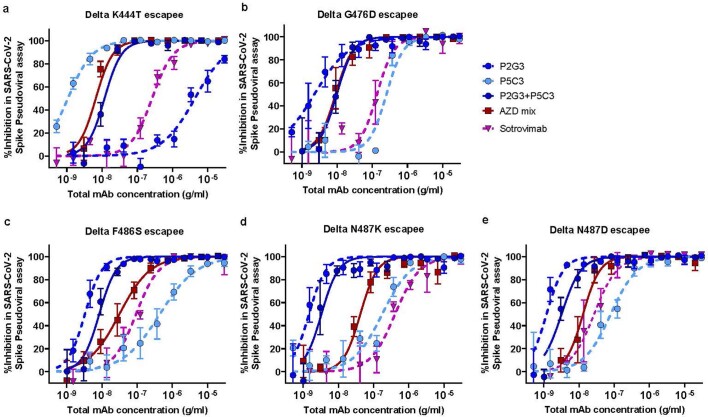
P2G3 and P5C3 efficiently cross-neutralize Spike-coated pseudoviruses with mutations encoding one another’s escapees. Neutralization of lentiviral particles pseudotyped with Delta variant SARS-CoV-2 Spike encoding: **a**) the P2G3-escaping K444T Spike substitution and P5C3-escaping **b**) G476D **c**) F486S, **d**) N487K and **e**) N487D Spike substitutions. Results shown consist of n = 6 replicates for P2G3 and P2G3 + P5C3, n = 5 or 6 replicates for P5C3 and the AZD mix and n = 3–5 replicates for Sotrovimab. Mean values ± SEM are shown. Source data

Monoclonal antibodies, as other classes of antivirals, are typically used in combination to prevent the emergence of resistant viruses. To gain insight into the predicted clinical value of our mAbs, we characterized the emergence of mutants escaping their blockade in tissue culture. For this, we grew SARS-CoV-2 Delta and Omicron BA.1 variants in the presence of suboptimal neutralizing doses of either P2G3 or P5C3 for three passages to generate a heterogeneous viral population, before switching to stringent mAb concentrations to select bona fide escapees (Fig. 6a). Viral genome sequencing of these mAb-resistant mutants pointed to the importance of spike substitutions G476D, F486S and N487K/D/S for escaping P5C3, and K444T for avoiding P2G3 neutralization. We thus tested the impact of these mutations on viral infectivity using lentivector pseudotypes. P5C3-escaping spike proteins were significantly less infectious than the wild-type control in both the ancestral D614G and Delta backgrounds, correlating with a drop in affinity for the viral ACE2 receptor in an in vitro binding assay, whereas the P2G3-escaping K444T substitution had a milder effect in both assays **(**Fig. 6b,c**)**. Yet, an examination of the GISAID EpiCoV database revealed that mutations G476D, F486S or N487K/D/S found in P5C3 escapees are only exceptionally encountered in SARS-Cov-2 isolates, together representing only 0.0087% of the 8,568,006 available sequences as of March 2022, and that the K444T mutation is equally rare (0.0024% of compiled sequences), strongly suggesting that the corresponding viruses have a markedly reduced fitness in the wild. Moreover, cross-neutralization studies demonstrated that P2G3 completely blocked the infectivity of P5C3-escaping Delta derivatives (Fig. 6d), and P5C3 and P2G3 efficiently cross-neutralized each other’s escape mutants in the Delta backgrounds (Extended Data Fig. 10).

## Discussion

We report the identification and characterization of P2G3, an anti-SARS-CoV-2 mAb with superior breadth and potency that can neutralize all VOCs so far, including the recently identified Omicron BA.1 and BA.2 variants. Structural and competitive binding studies demonstrate that P2G3 is a Class 3 mAb that recognizes an epitope on the RBD different from those bound by all other therapeutic mAbs authorized or at an advanced stage of development^8,10,11^. Sequence analyses also revealed that the P2G3 HCDR3 presents only 55% identity with any of the 4,897 anti-spike HCDR3 described thus far.

Cryo-EM revealed that P2G3 can bind both the up- and down-RBD conformations of the spike trimer, burying a large surface area of around 700 Å^2^ in a highly conserved region of RBD. Importantly, the binding epitope is largely non-overlapping with residues mutated in Omicron, a feature unique among almost all potent anti-SARS-CoV-2 mAbs reported so far^11^. The S371L Omicron mutation alone remarkably reduces neutralization activity of multiple potent mAbs of different binding classes despite not being included in their footprint^11^, perhaps because local conformational changes in the 370–375 loop impact the up- and down-RBD states and/or interfere with the critical gate opener N343 glycan positioning^30^; yet, this mutation is without effect on P2G3 neutralization. The unique angle of attack of P2G3 on the RBD domain, predicted to allow binding in both up- and down-RBD positions of the spike trimer, may explain why this antibody remains potently active against the Omicron variant.

We predicted that S309/Sotrovimab is also sterically free to bind all RBDs within the Omicron trimer, but found that this antibody had a marked loss of activity against this variant. Although the epitope bound by P2G3 partly overlaps with the region recognized by S309/Sotrovimab, the latter mAb does not block the RBD/ACE2 interaction, and has been proposed to act by alternative mechanisms including the inhibition of cell adhesion through C-type lectins^22^. Therefore, the improved binding affinity of P2G3 combined with the additional inhibitory effect on RBD/ACE2 interaction helps explain its 10 to 60-fold improved activity across all VOCs as compared with S309-Sotrovimab. Apart from the in vitro neutralization activity, P2G3 alone and/or in combination with P5C3 confers an excellent in vivo protection in the SARS-CoV-2 Omicron non-human primate (NHP) challenge model in both the prophylactic and therapeutic settings. Our NHP data also confirm studies in mice and hamsters indicating that the Omicron variant has reduced proliferation capacity in several animal models compared with other VOCs^31,32^.

Despite the exceptional neutralization profile of P2G3 against currently circulating variants, development of resistance is inevitable when a virus is under selective pressure. We report here that P5C3, a previously described potent and broadly active neutralizing mAb that acts by blocking the spike-ACE2 interaction, not only targets a highly conserved region of the RBD but can also bind Omicron spike concomitantly with P2G3. We further identify mutants capable of escaping neutralization by either one of these mAbs, but demonstrate that they have reduced infectivity, are extremely rare in the wild (suggesting reduced fitness) and can efficiently neutralize each other’s escapees, thereby validating their use in combination. It is noteworthy that P5C3 and P2G3 both neutralize the now prevalent R346K-containing BA.1.1 and BA.2 Omicron sub-variants, which partly or completely escape blockade by other currently available mAbs. Furthermore, the emerging Omicron BA.4 and BA.5 variants with RBD mutations L452R and F486V are distant from the P2G3 binding epitope and unlikely to impact neutralizing activity^2^.

Many of the advances gained so far in the COVID-19 pandemic, including COVID-19 vaccines and potent neutralizing mAbs, were largely eroded within months by the emergence of the Delta and Omicron variants. The Omicron variant exhibits markedly reduced sensitivity to vaccine-induced humoral immunity in healthy donors and, importantly, immunocompromised individual are now almost completely unprotected due to their inability to mount a protective humoral immune response following vaccination^33^. For these vulnerable individuals, we propose that passive immunization with 2–3 injections per year with the extended half-life P2G3 and P5C3 LS mAbs^27^ that can simultaneously bind to highly conserved and distinct epitopes on the viral spike, might represent an attractive prophylaxis option^34^. With potent neutralization, Fc-mediated functional activity and demonstrated in vivo protection, this broadly active combination, subject to successful development and authorization, has the potential to be a superior anti-SARS-CoV-2 mAb cocktail for prophylactic and therapeutic interventions against all current VOCs, and its breadth of activity suggests that it might be capable of neutralizing many future SARS-CoV-2 VOCs.

## Methods

### Study COVID-19 donors

Serum and blood mononuclear cell samples were from donors participating in the ImmunoCov and ImmunoVax studies performed by the Immunology and Allergy Service, Lausanne University Hospital, with all participants being adults of varying ages and having signed informed consent forms for the use of biological samples. Study design and use of subject samples were approved by the Institutional Review Board of the Lausanne University Hospital and the ‘Commission d’éthique du Canton de Vaud’ (CER-VD with trial reference numbers 2020-00620 and 2021-00041, respectively).

### Production of SARS-CoV-2 spike proteins

SARS-Cov-2 spike mutations are similar for all the cloned spike variants and the corresponding viral isolates, and are listed in Supplementary Table 2. Production of 2019-nCoV (D614G), B.1.17, B.1.351 and P.1 variants has been previously described^21^. RNA isolated from an anonymized leftover sample of an individual suspected to be SARS-CoV-2 Omicron strain-infected was reverse transcribed into complementary DNA. The Omicron spike ectodomain was amplified by PCR with primers (listed in Supplementary Table 3) designed on consensus sequence from available Omicron sequences, and introduced by in-fusion cloning into the nCoV-2P-F3CH2S plasmid, replacing the original wild-type spike^29^. The 2 prolines (P986–P987) and the furin cleavage site mutations (residues 682–685 mutated to GSAS) stabilizing the spike protein in the trimeric prefusion state were further introduced simultaneously by PCR and in-fusion-mediated site-directed mutagenesis using primers listed in Supplementary Table 3 as previously described^2^, and the full Omicron open reading frame (ORF) was sequence verified. The Delta B1.617.2 variant clone was generated by gene synthesis with a codon-optimized spike ORF (GenScript). The final constructs encode the spike ectodomains containing a native signal peptide, the 2P and furin cleavage site mutations, a C-terminal T4 fold-on fusion domain to stabilize the trimer complex, followed by C-terminal 8x His and 2x Strep tags for affinity purification. The trimeric spike variants were produced and purified as previously described^16^. The purity of Omicron spike trimers used for cryo-EM was determined to be >99% by SDS–PAGE analysis. Biotinylation of spike or RBD proteins was performed using EZ-Link NHS-PEG4-biotin (Life Technologies) with a 3-fold molar excess of reagent following the manufacturer’s protocol. Biotinylated proteins were buffer exchanged with PBS using an Amicon Ultra-0.5 with a 3 kDa molecular weight cut-off. Spike and RBD tetramers were prepared fresh before use and formed by combining biotinylated proteins with PE-conjugated streptavidin (BD Biosciences) at a molar ratio of 4:1.

### Binding and ACE2 blocking studies with SARS-CoV-2 spike

Luminex beads used for the serological and purified antibody binding assays were prepared by covalent coupling of SARS-CoV-2 proteins with MagPlex beads using a Bio-Plex amine coupling kit (Bio-Rad) following the manufacturer’s protocol. Each of the SARS-CoV-2 spike proteins expressed with different mutations were coupled with different coloured MagPlex beads so that tests could be performed with a single protein bead per well or in a multiplexed Luminex binding assay. Binding curves for antibody affinity measurements and the spike-ACE2 interaction assay were performed as previously described^16,35^ using anti-human IgG-PE secondary antibody (OneLambda ThermoFisher; H10104; 1:100 dilution) for antibody detection in spike Luminex binding assay and anti-mouse IgG-PE secondary antibody (OneLambda ThermoFisher; P-21129; 1:100 dilution) in the spike-ACE2 surrogate neutralization assay. Competitive binding studies were performed by pre-incubating 25 µg ml^−1^ of the indicated competitor antibody with the original 2019-nCoV RBD protein-coupled Luminex beads for 30 min. Biotinylated P5C3, P2G3, REGN10933, REGN10987, AZD8895, AZD1061, ADG-2 or S309 antibodies (prepared as described above) were added to each well at 1 µg ml^−1^, followed by a further 20 min incubation. Biotinylated antibody bound to RBD in the presence of competitor was stained with streptavidin-PE at a 1:1,000 dilution (BD Biosciences) and analysed on a 200 Bioplex instrument. COVID-19 serum samples from >100 donors were monitored for levels of IgG antibody binding to the SARS-CoV-2 spike trimer proteins from 2019-nCoV, D614G, Alpha, Beta and Gamma variants in the Luminex bead-based assay.

### Anti-spike B-cell sorting, immortalization and cloning

Blood from ImmunoVax study donors were collected in EDTA tubes and isolation of blood mononuclear cells was performed using Leucosep centrifuge tubes (Greiner Bio-one) pre-filled with density gradient medium (Ficoll-Paque PLUS, GE Healthcare) according to the manufacturer’s instructions. Freshly isolated cells were stained with the cocktail of fluorescent conjugated antibodies containing mouse anti-human CD19 APC-Cy7 (BD Biosciences; 557791; clone SJ25C1; 5 µl titration), mouse anti-human CD3-BV510 (BD Biosciences; 563109; cClone UCHT1; 1 µl titration), mouse anti-human IgM-FITC (Biolegend; 314506; clone MHM-88; 2 µl titration), mouse anti-human IgD PECF594 (BD Biosciences; 562540; clone IA6-2; 3 µl titration), mouse anti-human CD27-APC (BD Biosciences; 558664; clone M-T271; 5 µl titration) and mouse anti-human CD38-V450 (BD Biosciences; 646851; clone HB7; 5 µl titration) mAbs were used for antigen specific B-cell sorting, along with the pre-complexed Beta variant spike tetramer (2 µg in 100 µl) coupled to PE-streptavidin (BD Biosciences; SA10044; 4:1 molar ratio). All other aspects of cell sorting, immortalization protocol using Epstein Barr virus-positive (EBV) supernatants from B95-8 cells and cloning were as described in Fenwick et al.^21^. Sequences for mAbs P2G3 and P5C3 are provided in PDB submissions 7QTI, 7QTK and 7QTJ.

### SARS-CoV-2 live virus stocks

All biosafety level 3 procedures were approved by the Swiss Federal Office of Public Health. The SARS-Cov-2 D614G isolate and B.1.1.7 clone have previously been described^21^. Beta (EPI_ISL_981782), Gamma (EPI_ISL_981707), Delta B1.617.2 (EPI_ISL_1811202) and Omicron B.1.1.529.1 (EPI_ISL_7605546) early isolates were a kind gift from I. Eckerle, Geneva University Hospitals. Viral stocks were prepared in EPISERF medium on Vero E6 or Calu-3 (for Omicron) cells, aliquoted, frozen and titrated on Vero E6 cells.

### SARS-CoV-2 live virus cell-based cytopathic effect neutralization assay

Neutralization assay was performed as previously described, except for Delta and Omicron isolates where EPISERF medium instead of DMEM with 2% FCS was used to prepare antibody serial dilutions. Equal amounts of different viruses were used in all experiments (1,200 plaque forming units per well), except for the less cytopathic Omicron strain, where 2.5 times more virus was incubated with each antibody tested in parallel.

### Selection of resistant virus in the presence of mAbs

The day before infection, 293T + ACE2 (±TMPRSS2) cells were seeded in 6-well plates coated with poly-lysine at a density of 1 × 10^6^ cells per well. To generate a viral population under mAb pressure, early passage virus was diluted in 1 ml EPISERF with 2% FCS and incubated with 0.25 ng ml^−1^ mAb for 1 h at 37 °C in duplicates. Each mixture was added to the cells and P1 (passage 1) supernatants were collected 3 d later and clarified on 0.45 um SpinX filters centrifuged at 4,000 × *g* for 4 min. Aliquots of cleared P1 supernatants were diluted 1:40 in 2% DMEM, incubated with mAbs as described above and used to infect fresh cells for 4 d. P2 supernatants were treated as P1, and P3 supernatants were collected for RNA extraction and the subsequent selection step. To select for mAb-resistant viruses, 100 µl of the cleared undiluted P3 heterogeneous viral population was incubated with 100 µl mAbs at 2.5 µg ml^−1^, 0.625 µg ml^−1^ or 0.155 µg ml^−1^ final concentration for 1 h at 37 °C. The mixture was then applied on cells in 400 µl 2% DMEM (1:2 volume) for 3–4 d. Viruses were propagated for a few more passages and aliquots of each passage were used for RNA extraction and sequencing. Virus produced in the absence of mAb was collected and treated the same way in parallel to control for the appearance of mutations due to cell culture conditions.

### Spike-pseudotyped lentivectors production and neutralization assays

HDM-IDTSpike-fixK plasmid (BEI, NR-52514; obtained from J. D. Bloom, Fred Hutchinson Cancer Research Center) was used as backbone for all the clonings. For Alpha and Beta clones, the HDM-IDTSpike-fixK NotI/SmaI fragments were swapped with the respective Alpha and Beta fragments from the previously described pTwist plasmids^21^. Alpha P681H, T716I, S982A, D1118H and Beta A701V were further added in respective ORFs as well as R346K in the D614G plasmid by in-fusion-mediated site-directed mutagenesis using primers described in Supplementary Table 3. Delta B1.617.2 clone was generated by gene synthesis with a codon-optimized spike ORF (GenScript). The Omicron ORF was amplified from an RNA as described for protein production, with primers listed in Supplementary Table 3. Pseudoviruses were alternatively produced with the original 2019-nCoV (100976), Alpha/B.1.1.7 (101023) and Beta/B.1.351 (101024) pCAGGS-SARS2-spike vectors obtained from NIBSC. These vectors were co-transfected with pMDL p.RRE, pRSV.Rev and pUltra-Chili-Luc vectors (Addgene) into HEK 293T cells in DMEM medium+10% FCS using Fugene 6 (Promega) for pseudoviruses production. Neutralization assays were performed as previously described^21^.

### NHP challenge model for SARS-CoV-2 Omicron BA.1 infection

Cynomolgus macaques (*Macaca fascicularis*) originating from Mauritian AAALAC certified breeding centres were used in this study. All animals were housed within IDMIT animal facilities at CEA, Fontenay-aux-Roses under BSL-2 and BSL3 containment when necessary (Animal facility authorization no. D92-032-02, Préfecture des Hauts de Seine, France) and in compliance with European Directive 2010/63/EU, French regulations and the Standards for Human Care and Use of Laboratory Animals of the Office for Laboratory Animal Welfare (OLAW, assurance no. A5826-01, U.S.A.). Animals tested negative for *Campylobacte*r, *Yersinia*, *Shigella* and *Salmonella* before being used in the study.

The protocols were approved by the institutional ethical committee ‘Comité d’Ethique en Expérimentation Animale du Commissariat à l’Energie Atomique et aux Energies Alternatives’ (CEtEA no. 44) under statement number A20-011. The study was authorized by the ‘Research, Innovation and Education Ministry’ under registration number APAFIS 24434-2020030216532863.

In the prophylactic protection study, four female cynomolgus macaques aged 3–6 years were randomly assigned between the control and treated groups to evaluate the efficacy of P2G3 LS in protecting from challenge with the SARS-CoV-2 Omicron BA.1 virus. The treated group (*n* = 2 (MF1 and MF2)) received one dose at 10 mg kg^−1^ of P2G3 LS human IgG1 monoclonal antibody delivered by intravenous slow bolus injection over 3–8 min 3 d before challenge, while control animals (*n* = 2 in parallel (MF3 and MF4)) and historical animals (*n* = 2 (MF5 and MF6)) received no treatment. The limited number of animals used was a first exploratory evaluation of P2G3 LS efficacy in the NHP model. In the therapeutic study, 16 female cynomolgus macaques aged 3–6 years were randomly assigned between the control untreated (*n* = 4), the 2.5 mg kg^−1^ P2G3 LS + 2.5 mg kg^−1^ P5C3 LS (*n* = 6) and the 5 mg kg^−1^ P2G3 LS + 5 mg kg^−1^ P5C3 LS (*n* = 6) treated groups to evaluate the efficacy of the P2G3 LS/P5C3 LS combination in the suppression and elimination of the SARS-CoV-2 Omicron BA.1 virus. All animals were then exposed to a total dose of 10^5^ TCID_50_ of Omicron B.1.1.529 SARS-CoV-2 virus produced in Calu-3 cells (NIH/BEI reference: NR-56462) via the combination of intranasal and intratracheal routes (day 0 in the prophylactic study and 24 h in the therapeutic study), with sample collection and testing performed as previously described^36^. Tracheal swabs, nasopharyngeal swabs and bronchoalveolar lavages were performed on all NHPs throughout the study to monitor levels of both genomic and subgenomic RNA for the SARS-CoV-2 virus. Blinding was performed whereby the technician analysing samples for RNA and virus titration was not aware of the treatment groups being evaluated and all animals and data points were included in the analysis. The NHP sample size was selected on the basis of the large, 1- to 2-log reduction in viral RNA anticipated in the tracheal, nasopharyngeal and/or BAL samples, with an effective therapy that can provide statistically significant differences between treated and untreated NHPs. These sample size assumptions were confirmed with the statistical differences observed in viral RNA levels evaluated using the Mann-Whitney two-sided tests to compare control and treatment groups.

### Hamster challenge model SARS-CoV-2 infection

KU LEUVEN R&D has developed and validated a SARS-CoV-2 Syrian golden hamster infection model that is suitable for the evaluation of potential antiviral activity of novel antibodies^37–39^. The SARS-CoV-2 strain used in this study, BetaCov/Belgium/GHB-03021/2020 (EPI ISL 109407976|2020-02-03), was recovered from a nasopharyngeal swab taken from an RT–qPCR-confirmed asymptomatic patient who returned from Wuhan, China in the beginning of February 2020. A close relation with the prototypic Wuhan-Hu-1 2019-nCoV (GenBank accession 11 no. MN908947.3) strain was confirmed by phylogenetic analysis. Infectious virus was isolated by serial passaging on HuH7 and Vero E6 cells^37^; passage 6 virus was used for the study described here. The titre of the virus stock was determined by endpoint dilution on Vero E6 cells by the Reed and Muench method. Live virus-related work was conducted in the high-containment A3 and BSL3+ facilities of the KU Leuven Rega Institute (3CAPS) under licences AMV 30112018 SBB 219 2018 0892 and AMV 23102017 SBB 219 20170589 according to institutional guidelines.

The hamster infection model of SARS-CoV-2 has been previously described^37,39^. The animals were acclimated for 4 d before study start. Housing conditions and experimental procedures were approved by the ethics committee for animal experimentation of KU Leuven (license P065-2020). Female hamsters (6–8 weeks old) were administered IgG1 isotype control (5 mg kg^−1^), P2G3 LS (5 mg kg^−1^, 1 mg kg^−1^ or 0.5 mg kg^−1^) or REGN10933 (5 mg kg^−1^) by intraperitoneal injection. Two days later, hamsters were anaesthetized with ketamine/xylazine/atropine, blood samples were collected and animals were inoculated intranasally with 2.4 × 10^6^ median TCID_50_ of SARS-CoV-2 (day 0). Hamsters were monitored for appearance, behaviour and weight. Antibody concentrations present in the hamster plasma on day 0 of the study were measured using the Luminex assay described above with spike trimer-coupled beads and using purified P2G3 LS antibody to generate a standard curve. In these studies, no control animals were excluded. In treated groups, animals with undetectable levels of serum antibodies (2 hamsters in 5 mg kg^−1^ P2G3 LS group, 1 hamster in 1 mg kg^−1^ P2G3 LS group and 1 hamster in 5 mg kg^−1^ REGN10933 group) were excluded from the analysis as this indicated a technical failure in the drug administration. At day 4 post infection, hamsters were killed and lung tissues were homogenized using bead disruption (Precellys) in 350 μl TRK lysis buffer (E.Z.N.A. total RNA kit, Omega Bio-tek) and centrifuged (10,000 r.p.m., 5 min) to pellet the cell debris. RNA was extracted according to the manufacturer’s instructions. Of 50 μl eluate, 4 μl was used as a template in RT–qPCR reactions. RT–qPCR was performed on a LightCycler96 platform (Roche) using the i*Taq* Universal Probes one-step RT–qPCR kit (Bio-Rad) with N2 primers and probes targeting the nucleocapsid^37^. Standards of SARS-CoV-2 cDNA (IDT) were used to express viral genome copies per mg tissue. For endpoint virus titrations, lung tissues were homogenized using bead disruption (Precellys) in 350 μl minimal essential medium and centrifuged (10,000 r.p.m., 5 min, 4 °C) to pellet the cell debris. To quantify infectious SARS-CoV-2 particles, endpoint titrations were performed on confluent Vero E6 cells in 96-well plates. Viral titres were calculated by the Reed and Muench method using the Lindenbach calculator and were expressed as TCID_50_ per mg tissue. The hamster sample size was selected on the basis of the large, >1-log reduction in viral RNA and infectious virus anticipated in the lung tissue with an effective therapy that can provide statistically significant differences between treated and untreated animals. These sample size assumptions were confirmed in our statistical analysis. Statistical differences in viral RNA levels and infectivity were evaluated using the Mann-Whitney two-sided tests to compare control and treatment groups.

### Antibody Fc-mediated functional activity assays

ADCC and ADCP assays were performed as previously described with minor changes^40^. Cryopreserved peripheral blood mononuclear cells (PBMCs) from healthy patients were thawed and resuspended at 1 million ml^−1^ in RPMI medium (Gibco, Life Technologies) supplemented with 10% heat-inactivated fetal bovine serum (FBS), 100 IU ml^−1^ penicillin and 100 µg ml^−1^ streptomycin (BioConcept). Cells were stimulated with 25 ng ml^−1^ IL-15 (Miltenyi Biotec) for 6 h and ADCC effector cells were enriched from PBMCs by depletion of T cells using anti-CD3-coupled magnetic beads from the EasySep human T-cell isolation kit (Stemcell). Target cells used for the ADCC assay were a CEM-NK resistant cell line that was stably transfected to express the original 2019-nCoV spike protein at the cell surface and with constitutive expression of the Luciferase gene (CEM-NKR-Spike-Luc cells). In the ADCC assay, CEM-NKR-Spike-Luc cells were incubated with anti-spike antibody at 0.3 µg ml^−1^, isotype control antibodies at 0.3 µg ml^−1^ or an anti-HLA class I (MHC) positive control antibody (Invivogen) at 0.005 µg ml^−1^. Following 5 min at room temperature, the CEM-NKR-Spike-Luc cells/antibody mixtures were then co-cultured overnight at a 1:10 ratio of CD3-depleted PBMC effector cells in RPMI medium supplemented with 5% low-IgG FBS and 1% penicillin-streptomycin in U-bottom 96-well plates (Sarstedt). The following day, cell killing was monitored either directly by flow cytometry or indirectly by monitoring the decrease in luciferase activity associated with cell death. In flow cytometry analysis, spike-transfected CEM-NKR-Luc cells were stained with PKH26 kit according to the manufacturer's protocol (Sigma; MINI26-1KT) before performing the ADCC assay. To monitor cell killing, co-cultured cells were washed and stained with fluorescent conjugated antibodies, mouse anti-human CD56-AF488 (BD Biosciences; 557699; clone B159; 5 µl titration), mouse anti-human CD16-FITC (BD Biosciences; 555406; clone 3G8; 2 µl titration) and mouse anti-human CD4-PECF594 (BD Biosciences; 5562316; clone RPA-T4; 2 µl titration). Annexin V-APC (Invitrogen; 88-8102-72; 2 µl titration) and Aqua Live/Dead cell stain (Invitrogen; L34966; 1:400 dilution) were used for ADCC validation tests, anti-HLA class I antibody was used as positive control (Invivogen; MA1-19027; clone W6/32; 20 ng ml^−1^) and cells were then analysed using a FACS LSR II cytometer instrument with Diva software v6.1.2. Spike CEM-NKR-Luc cells were gated with the PKH26 fluorochrome and then dead (Aqua positive) and apoptotic/dying (Annexin V positive) cells were evaluated for the positive (anti-HLA class I), negative (isotype control) and test (anti-spike antibodies) antibody conditions to establish ADCC activity. In the luciferase readout assay, co-cultured cells were transferred in white-Elmer 96-well plates and luciferase activity was measured using a one-step luciferase assay kit (BPS Biosciences) on a Synergy plate reader. Co-cultured spike CEM-NKR-Luc cells incubated with isotype control antibodies generally gave 5–10% reduced luminescence signal compared with CEM-NKR-Luc cells incubated in the absence of effector cells. Positive control anti-MHC (anti-HLA class I) antibody gave strong antibody-dependent cell killing of 60–80% and anti-spike antibodies gave intermediate responses.

For the ADCP assay, TransFluoSpheres carboxylate-modified microspheres (1.0 µm diameter, 488 nm/560 nm excitation/emission; ThermoFisher) were coupled directly with 2019-nCoV trimeric spike protein or with streptavidin according to the manufacturer's protocol. Spike-coupled beads were washed, incubated in the presence or absence of the different concentrations of anti-spike antibody for 30 min and then the mixture was added directly to the U937 monocyte cell line plated in a U-bottom 96-well plate (Sarstedt). Following an overnight incubation, cells were analysed using a FACS LSR II cytometer to identify cells with spike bead fluorescence. U937 cells incubated with spike beads in the absence of antibody generally showed <5% phagocytic activity, while increased ADCP activity was observed with increasing concentration of anti-spike antibody with a maximum of 100% of cells exhibiting fluorescence associated with spike bead phagocytosis. ADCP activity of Omicron spike-coated beads was measured by pre-incubating streptavidin-coupled TransFluoSpheres beads with biotinylated Omicron spike protein, produced as described above. Beads were washed after 30 min and used in the ADCP assay as described for the directly coupled 2019-nCoV trimeric spike beads.

### Cryo-EM

Cryo-EM grids were prepared with a Vitrobot Mark IV (ThermoFisher). Quantifoil R1.2/1.3 Au 400 holey carbon grids were glow-discharged for 120 s at 15 mA using a PELCO easiGlow device (Ted Pella). Omicron spike (3.0 µl, 0.7 mg ml^−1^) mixed with 0.16 mg ml^−1^ each of P5C3 and P2G3 Fab fragments (final ratio of 3.2 uM Omicron spike:1.5 uM P5C3:1.5 uM P2G3) was applied to the glow-discharged grids, blotted for 6 s under blot force 10 at 100% humidity and 4 °C in the sample chamber, and the blotted grid was plunge-frozen in liquid nitrogen-cooled liquid ethane.

Grids were screened for particle presence and ice quality on a TFS Glacios microscope (200 kV), and the best grids were transferred to a TFS Titan Krios G4. Cryo-EM data were collected using a TFS Titan Krios G4 transmission electron microscope, equipped with a Cold-FEG on a Falcon IV detector in electron counting mode. Falcon IV gain references were collected just before data collection. Data were collected using TFS EPU v2.12.1 utilizing the aberration-free image shift protocol, recording 4 micrographs per ice hole.

Movies were recorded at a magnification of ×165,000, corresponding to the 0.83 Å pixel size at the specimen level, with defocus values ranging from −0.8 to −2.5 µm. Exposures were automatically adjusted to 60 e^−^ Å^−2^ total dose, resulting in an exposure time of approximately 3 s per movie. In total, 22,758 micrographs in EER format were collected.

### Cryo-EM image processing

On-the-fly processing was first performed during data acquisition to evaluate data quality during screening by using cryoSPARC live v3.3.1^41^. The obtained ab-initio structures were used for better particle picking for template creation. Motion correction was performed on raw stacks without binning, using the cryoSPARC implementation of motion correction^42^. Particles (1,454,045) were automatically template-picked. Three rounds of two-dimensional (2D) classification were performed, resulting in a particle set of 383,541 particles. Selected particles resulting from the 2D classification were used for ab-initio reconstruction and hetero-refinement. After hetero-refinement, 189,500 particles contributed to an initial 3D reconstruction of 2.79 Å resolution (Fourier-shell coefficient (FSC) 0.143) with C1 symmetry. These particles were subjected to 3D classification resulting in 10 classes. Class 9 resulted in a global map of the Omicron spike with an RBD-up bound to a P5C3 and P2G3 Fab at a resolution of 3.04 Å (FSC 0.143) with C1 symmetry. Focused refinement of Class 4 with a soft mask volume encompassing an RBD-up and its bound Fab and an adjacent RBD-down resulted in a map at 4.01 Å (FSC 0.143) with C1 symmetry. Finally, focused refinement of Class 5 with a soft mask volume encompassing an RBD-down and its bound P2G3 and an adjacent N-terminal domain (NTD) resulted in a map at 3.84 Å (FSC 0.143) with C1 symmetry. The soft mask volumes were generated manually in UCSF Chimera and cryoSPARC^43^.

### Cryo-electron microscopy model building

A model of a spike trimer (PDB ID 7QO7; https://www.rcsb.org/structure/7QO7) or AlphaFold2 (ColabFold implementation) models of the P5C3 and P2G3 Fabs were fit into the cryo-EM maps with UCSF Chimera. These docked models were extended and rebuilt manually with refinement, using Coot and Phenix^44,45^. Figures were prepared in UCSF Chimera, UCSF ChimeraX and Pymol^43^. Numbering of the full-length spike models within the global map is based on Omicron numbering. Numbering of models containing just the RBD within the local maps is based on wild-type numbering. Fab numbering at one from the first constant domains for the heavy (CH1) and light chains (CL), respectively. Buried surface area measurements and centroid measurements were calculated within ChimeraX.

### Statistical analysis

Statistical parameters including the exact values of *n*, the definition of center, dispersion and precision measures (mean or median ± s.e.m.) and statistical significance are reported in the figures and figure legends. Data were judged to be statistically significant when *P* < 0.05. In figures, asterisks denote statistical significance as calculated using the two-tailed non-parametric Mann-Whitney U test for two-group comparisons or the Kruskal-Wallis test with Dunn’s multiple-comparison correction. Analyses were performed in GraphPad Prism and Microsoft Excel.

### Reporting summary

Further information on research design is available in the Nature Research Reporting Summary linked to this article.

## Supplementary information

Supplementary Information Supplementary Figs. 1 and 2, and Tables 1–3.

Reporting Summary

Supplementary Data 1 Source data for the detection of subgenomic RNA in the NHP therapeutic efficacy study.

## Data Availability

All data supporting the findings of this study are available within the paper and in the Source Data. The reconstructed maps of the global Omicron spike with Fabs bound are available from the EMDB database, C1 symmetry, EMDB-14141. The atomic model for the full-length Omicron spike with Fabs bound is available from the PDB database, PDB-7QTI. The local focused-refinement map of the RBD-up with two Fabs bound is available from the EMDB database, EMDB-14142. The atomic model for the RBD-up with two Fabs bound in the locally refined map is available from the PDB database, PDB-7QTJ. The local focused-refinement map of the RBD-down with P2G3 Fab bound is available from the EMDB database, EMDB-14143. The atomic model for the RBD-down with P2G3 Fab bound in the locally refined map is available from the PDB database, PDB-7QTK. All plasmids made in this study are available from the corresponding authors upon request. Source data are provided with this paper.

## Source data

Source Data Fig. 1 Source data for generating inhibition curves for blocking of spike-ACE2 interaction.
Source Data Fig. 2 Source data for pseudoviral and live virus cytopathic effect neutralization assays.
Source Data Fig. 3 Source data for viral genomic and subgenomic RNA in the NHP challenge study.
Source Data Fig. 4 Source data for viral genomic and subgenomic RNA in the NHP therapeutic efficacy study.
Source Data Fig. 6 Source data for the characterization of viral escape mutations.
Source Data Extended Data Fig. 1 Source data for spike binding and spike-ACE2 blocking activity assays.
Source Data Extended Data Fig. 2 Source data for pseudoviral and live virus neutralization assays.
Source Data Extended Data Fig. 3 Source data for ADCC and ADCP Fc-mediated functional assays.
Source Data Extended Data Fig. 4 Source data for the detection of infectious virus and viral RNA in the hamster challenge model.
Source Data Extended Data Fig. 10 Source data for pseudoviral neutralization assays performed with spike encoding P2G3 and P5C3 escape mutations.
