# Supplementary material for: Patient-derived monoclonal antibody neutralizes SARS-CoV-2 Omicron variants and confers full protection in monkeys
Source: Nat Microbiol. 2022 Jul 25;7(9):1376–89. doi: 10.1038/s41564-022-01198-6 (PMC9418005; doi:10.1038/s41564-022-01198-6)
Supplement: Supplementary file 1 — Supplementary Figs. 1 and 2, and Tables 1–3. [file 41564_2022_1198_MOESM1_ESM.pdf]

nature portfolio

https://doi.org/10.1038/s41564-022-01198-6

Supplementary information

Patient-derived monoclonal antibody neutralizes SARS-CoV-2 Omicron variants and confers full protection in monkeys

In the format provided by the
authors and unedited

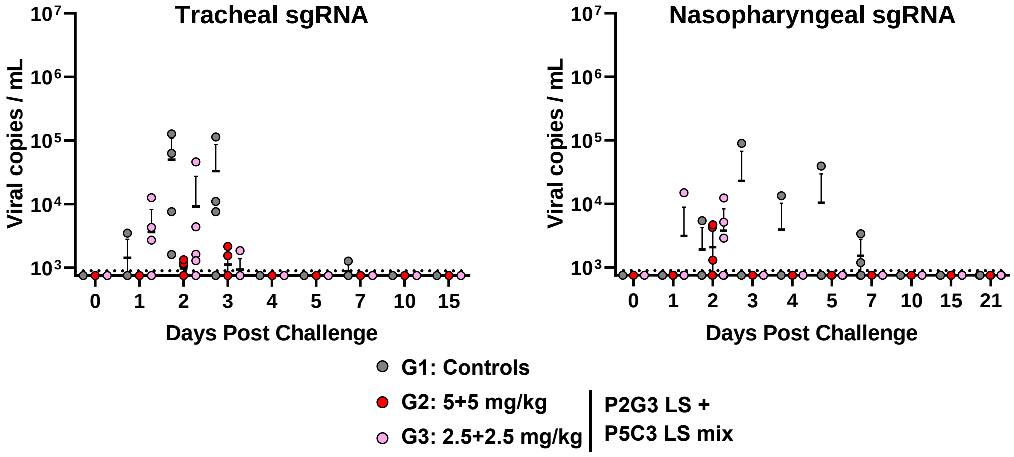

Supplementary Figure 1. - Post challenge therapeutic use of the P2G3 LS and P5C3 LS mAb combination suppresses detection of sgRNA in non-human primates challenged with the SARS-CoV-2 Omicron virus. Animals were challenged through intranasal and intratracheal inoculation of the Omicron BA.1 SARS-CoV-2 virus (1 x105 TCID50) on day 0 and 24 hours later, group 2 (G2, red circles, n=6) NHP were administered intravenous 5 mg/kg of P2G3 LS and 5 mg/kg of P2G3 LS, group 3 (G3, pink circles, n=6) were administered 2.5 mg/kg of P2G3 LS and 2.5 mg/kg of P2G3 LS and group 1 (G1, grey circles, n=4) were used as untreated reference controls. Tracheal swabs and nasopharyngeal swabs samples collected over the course of the study were evaluated for viral copies per ml of subgenomic RNA. Dotted line indicates lower limit of detection at 2.87-log copies per ml for viral sgRNA, respectively. Mean values ± SEM are shown.

RBD

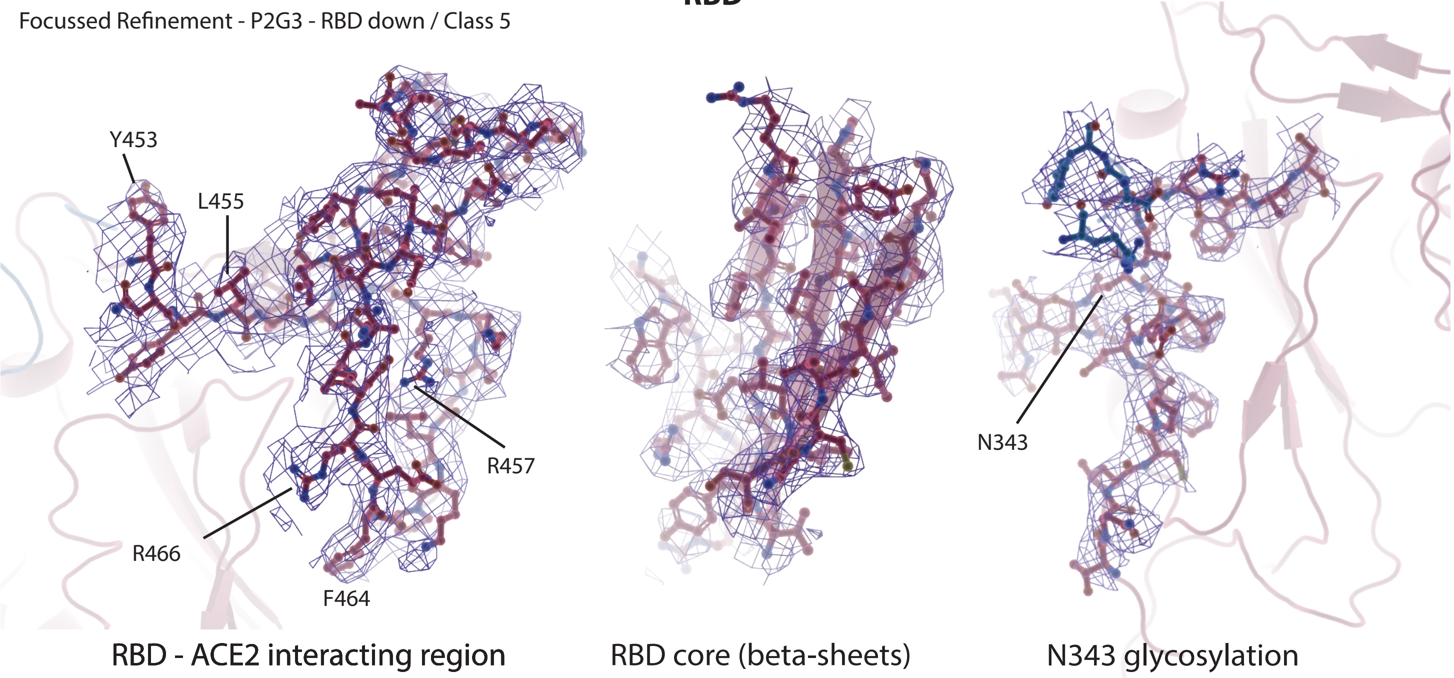

P2G3 Interacting Region

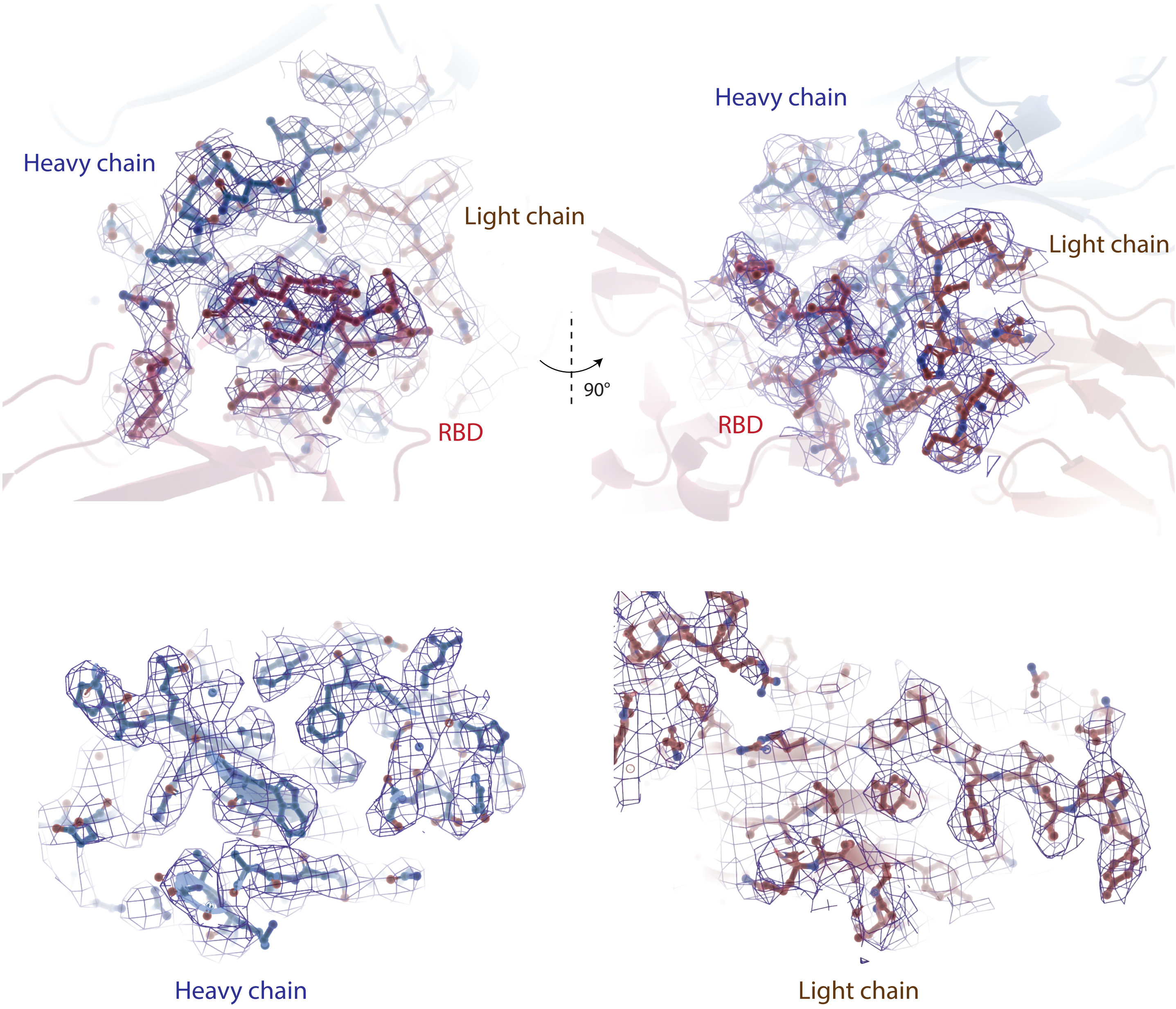

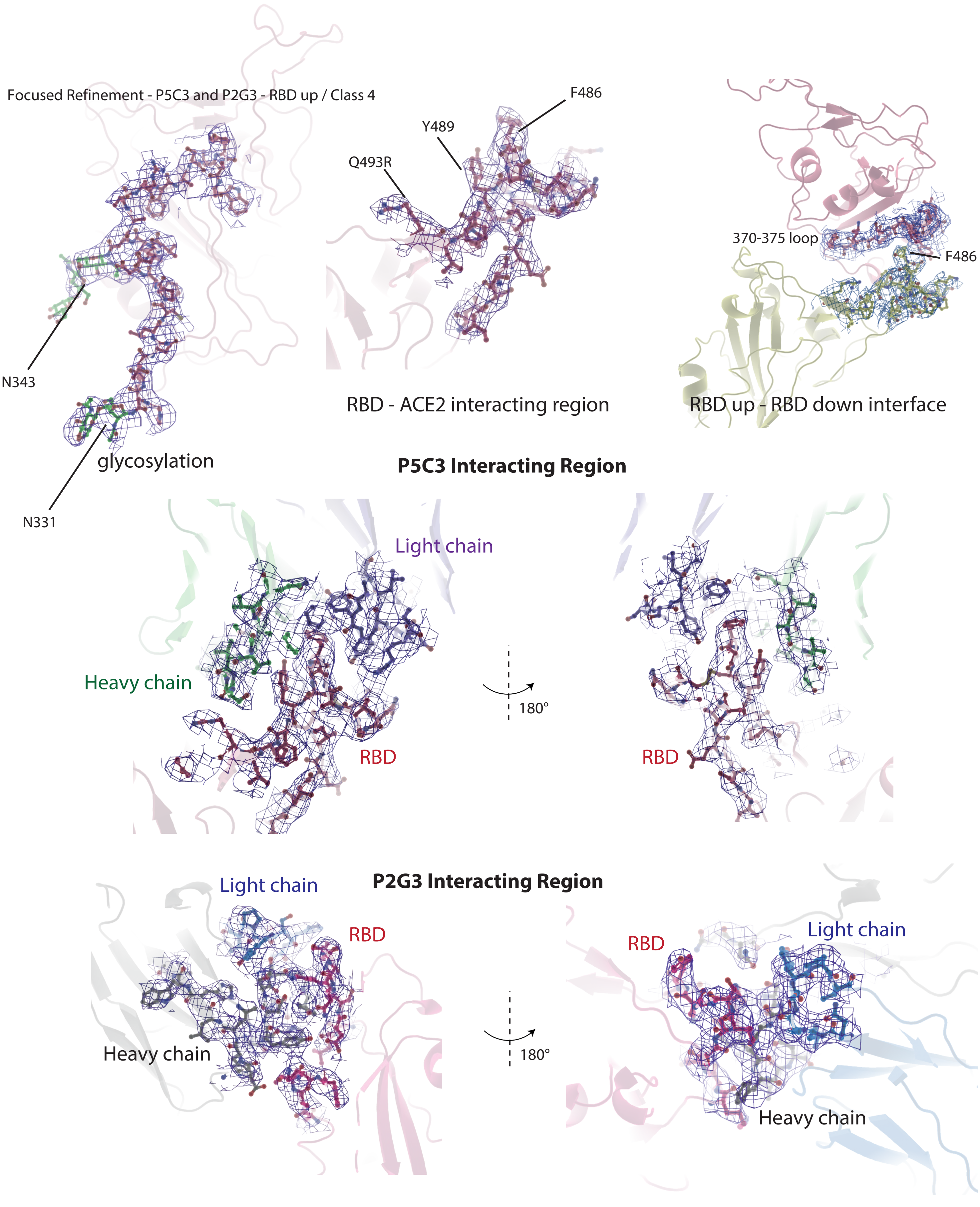

Supplementary Figure 2 - Highlights of regions of the Omicron Spike and Fabs with Cryo-EM density maps.
The Cryo-EM density is rendered as a mesh. The atomic model is shown as ribbon or stick representation.

Supplementary Data Table 1 - Cryo-EM data collection, refinement and validation statistics

	**SARS-CoV-2 S Omicron Spike B.1.1.529-P2G3-P5C3 (Global map)**	**SARS-CoV-2 S Omicron Spike B.1.1.529-P2G3 bound to RBD-down + RBD-up (Local map)**	**SARS-CoV-2 S Omicron Spike B.1.1.529-P2G3-P5C3 bound to RBD-up + RBD-down (Local map)**
**Microscope**	TFS Titan Krios G4 + E-CFEG		
**Detector**	Falcon 4		
Magnification	165kx		
Voltage (kv)	300		
Electron exposure (e-/Å²)	60		
Defocus range (um)	-0.8 to -2.5		
Pixel size (Å)	0.83		
Symmetry	C1		
Micrographs	22 758		
Initial particle images (No.) (After manual 2D class curation)	383 540		
Final particle images (No.)	28 478	21 672	18 839
Map resolution (Å) (*FSC 0.143*)	3.04	3.84	4.01
Map resolution range (Å)	25.9-2.76	10.5-3.02	30.0-3.02
**Refinement**			
Model resolution (Å) (*FSC 0.5*)	3.72	4.14	4.38
Initial model used	7QO7	---	---
Map sharpening B factor (Å²)	-24.4	-38.5	-44.1
Protein residues	5 061	643	1 280
**RMSD deviations**			
Bond lenths (Å) [#Z>5]	0.003 [0]	0.002 [0]	0.003 [1]
Bond angles (°) [#Z>5]	0.620 [16]	0.609 [2]	0.579 [4]
**Validation**			
**MolProbity score**	**1.82**	**1.85**	**1.77**
Clashscore	7.95	9.04	8.08
% Poor rotamers (%)	0.02	0.00	0.00
C-beta outliers (%)	0.00	0.00	0.00
**Ramachandran plot**			
Favored (%)	94.34	94.63	95.25
Allowed (%)	5.46	5.21	4.51
Disallowed (%)	0.20	0.16	0.24
**PDB**	7QTI	7QTK	7QTJ
**EMDB**	14141	14143	14142

Supplementary Table 2: Spike mutations

**Alpha B.1.1.7**	Δ69-70, Δ144, N501Y, A570D, D614G, P681H, T716I, S982A, D1118H
**Beta B.1.351**	L18F, D80A, D215G, Δ242-244, R246I, K417N, E484K, N501Y, D614G, A701V
**Gamma P.1**	L18F, T20N, P26S, D138Y, R190S, K417T, E484K, N501Y, D614G, H655Y, T1027I, V1176F
**Delta B.1.617.2**	T19R, Δ156-157, R158G, L452R, T478K, D614G, P681R, D950N
**Omicron B.1.1.529.1**	A67V, Δ69-70, T95I, G142D, Δ143-145, Δ211, L212I, ins214EPE, G339D, S371L, S373P, S375F, K417N, N440K, G446S, S477N, T478K, E484A, Q493R, G496S, Q498R, N501Y, Y505H, T547K, D614G, H655Y, N679K, P681H, N764K, D796Y, N856K, Q954H, N969K, L981F
**BA.2**	T19I, Δ24-26, A27S, G142D, V213G G339D, S371F, S373P, S375F, T376A, D405N, R408S, K417N, N440K, S477N, T478K, E484A, Q493R, Q498R, N501Y, Y505H, D614G, H655Y, N679K, P681H, N764K, D796Y, Q954H, N969K

Supplementary Table 3 : Primers used to clone spike mutations

HDM-IDT-S-Fix variants cloning for lentivectors pseudotypes

**Alpha**	O.HDM.1(f)	CTGGCCCATCACTTTGGCAAAG
	O.Ksprimer.2(b)	CGAGGTCGACGGTATCG
	O.T716I.1(f)	CAATAGCTATCCCAATAAATTTCACTATTTC
	O.T716I.2(b)	GAAATAGTGAAATTTATTGGGATAGCTATTG
	O.S982A.1(f)	GAATGATATCCTGGCACGGTTGGACAAG
	O.S982A.2(b)	CTTGTCCAACCGTGCCAGGATATCATTC
	O.D1118H.1(f)	ATTATTACCACCCACAATACCTTTG
	O.D1118H.2(b)	CAAAGGTATTGTGGGTGGTAATAAT
	O.del144 1(f)	CCTTCCTGGGCGTCTATCACAAGAAC
	O.del144 2(b)	GTTCTTGTGATAGACGCCCAGGAAGG
	O.A570D 1(f)	CTTTCCAACAATTCGGGCGGGACATAGATGATACCACTGACG
	O.A570D 2(b)	CGTCAGTGGTATCATCTATGTCCCGCCCGAATTGTTGGAAAG
**Beta**	O.HDM.1(f)	CTGGCCCATCACTTTGGCAAAG
	O.HDM.2(b)	GGCAGAATCTCAGTGGTGACCGAAATAGTG
	O.A701V.3(f)	GAGCCTCGGAGTAGAGAACAGCG
	O.A701V.4(b)	CGCTGTTCTCTACTCCGAGGCTC
**R346K**	O.R346K.3(f)	CTTCAATGCTACTAAATTCGCCTCAG
	O.R346K.4(b)	CTGAGGCGAATTTAGTAGCATTGAAG
**Omicron**	O.21K.1(f)	AGAATTCCGCGGGCGGCCGCCATGTTTGTTTTTCTTG
	O.21K.2(b)	ACGGTATCGATAAGCTTTATGTGTAATGTAATTTG
	O.21K.3(f)	GTCAACAACTCATATGAGTGTGA
	O.21K.4(b)	TCACACTCATATGAGTTGTTGAC

nCov S variants cloning for proteins purification

**Omicron**	O.21K.5(f)	AGGCCGAGTTCGGTACCGCCACCATGTTTGTTTTTCTTG
	O.21K.6(b)	CTCGGGGATGTATCCGGATCCTGCTCATACTTTCCAAG
	O.21K.7(f)	CACGTCTTGACCCTCCAGAGGCTGAAGTG
	O.21K.8(b)	CACTTCAGCCTCTGGAGGGTCAAGACGTG
	O.21K.9(f)	GACTAAGTCTCATGGGAGCGCAAGTAGTGTAGCTAGT
	O.21K.10(b)	ACTAGCTACACTACTTGCGCTCCCATGAGACTTAGTC
