# Supplementary material for: Patient-derived monoclonal antibody neutralizes SARS-CoV-2 Omicron variants and confers full protection in monkeys
Source: Nat Microbiol. 2022 Jul 25;7(9):1376–89. doi: 10.1038/s41564-022-01198-6 (PMC9418005; doi:10.1038/s41564-022-01198-6)
Supplement: Supplementary file 2 — Reporting Summary [file 41564_2022_1198_MOESM2_ESM.pdf]

nature portfolio

Corresponding author(s): Giuseppe Pantaleo and Didier Trono

Last updated by author(s): Jan 30, 2022

Reporting Summary

Nature Portfolio wishes to improve the reproducibility of the work that we publish. This form provides structure for consistency and transparency in reporting. For further information on Nature Portfolio policies, see our Editorial Policies and the Editorial Policy Checklist.

Statistics

For all statistical analyses, confirm that the following items are present in the figure legend, table legend, main text, or Methods section.

n/a	Confirmed	
☐	☒	The exact sample size (*n*) for each experimental group/condition, given as a discrete number and unit of measurement
☐	☒	A statement on whether measurements were taken from distinct samples or whether the same sample was measured repeatedly
☐	☒	The statistical test(s) used AND whether they are one- or two-sided<br>*Only common tests should be described solely by name; describe more complex techniques in the Methods section.*
☒	☐	A description of all covariates tested
☒	☐	A description of any assumptions or corrections, such as tests of normality and adjustment for multiple comparisons
☐	☒	A full description of the statistical parameters including central tendency (e.g. means) or other basic estimates (e.g. regression coefficient) AND variation (e.g. standard deviation) or associated estimates of uncertainty (e.g. confidence intervals)
☐	☒	For null hypothesis testing, the test statistic (e.g. *F*, *t*, *r*) with confidence intervals, effect sizes, degrees of freedom and *P* value noted<br>*Give P values as exact values whenever suitable.*
☒	☐	For Bayesian analysis, information on the choice of priors and Markov chain Monte Carlo settings
☒	☐	For hierarchical and complex designs, identification of the appropriate level for tests and full reporting of outcomes
☒	☐	Estimates of effect sizes (e.g. Cohen's *d*, Pearson's *r*), indicating how they were calculated

Our web collection on statistics for biologists contains articles on many of the points above.

Software and code

Policy information about availability of computer code

Data collection	Synergy H1 Hybrid Reader from Biotek, Gen5 V3.03 for luminescence readings<br>Bio-Plex 200 System from Bio-Rad, Bio-Plex Manager V6.1 from Bio-Rad for Luminex studies<br>LSR II BD with Diva Software v6.1.2 for flow cytometry analysis<br>TFS EPU v2.12.1 using aberration-free image shift protocol (AFIS), cryoSPARC live v3.3.1 for Cryo-EM
Data analysis	GraphPad Prism 9.1.0 was used to perform the statistical analysis along with MacSynergy II software<br>Flow cytotometry analysis was performed with FlowJo v10 or Cytobank software.<br>ImageJ 1.53e software woth the ReadPlate 3.0 Macro was used for densitometry analysis of plates in live virus CPE assays.<br>UCSF Chimera 1.16, UCSF Chimera X 1.3, Coot 0.8.9, Phenix 1.20.1-4887, Pymol 2.5, cryoSPARC v3.3.1-211214, Microsoft Excel 2016<br>No custom scripts were developed in this study.

For manuscripts utilizing custom algorithms or software that are central to the research but not yet described in published literature, software must be made available to editors and reviewers. We strongly encourage code deposition in a community repository (e.g. GitHub). See the Nature Portfolio guidelines for submitting code & software for further information.

Data

Policy information about availability of data

All manuscripts must include a data availability statement. This statement should provide the following information, where applicable:

- Accession codes, unique identifiers, or web links for publicly available datasets
- A description of any restrictions on data availability
- For clinical datasets or third party data, please ensure that the statement adheres to our policy

All data supporting the findings of this study are available within the paper and in the Source Data. The reconstructed maps of the global Omicron Spike with Fabs bound are available from the EMDB database, C1 symmetry, EMDB-14141. The atomic model for the full-length Omicron Spike with Fabs bound is available from the PDB database, PDB-7QTI. The local focussed-refinement map of the RBD-up with two Fabs bound is available from the EMDB database, EMDB-14142. The atomic model for the RBD-up with two Fabs bound in the locally refined map is available from the PDB database, PDB-7QTJ. The local focussed-refinement map of the RBD-down with P2G3 Fab bound is available from the EMDB database, EMDB-14143. The atomic model for the RBD-down with P2G3 Fab bound in the locally refined map is available from the PDB database, PDB-7QTK.

Field-specific reporting

Please select the one below that is the best fit for your research. If you are not sure, read the appropriate sections before making your selection.

☒ Life sciences ☐ Behavioural & social sciences ☐ Ecological, evolutionary & environmental sciences

For a reference copy of the document with all sections, see nature.com/documents/nr-reporting-summary-flat.pdf

Life sciences study design

All studies must disclose on these points even when the disclosure is negative.

Sample size	Sample size of four to six hamsters were used for each of the different antibody dosing groups. Given the multiple log reduction in the detection of infectious virus or viral RNA in the lung, this sample size was sufficient to see significant protection in all doses of P2G3 LS administered.<br>In the prophylactic protection study, four female cynomolgus macaques (Mauritian-origin) aged 3–6 years were randomly assigned between the control and P2G3 LS treated groups. Two historical control SARS-CoV-2 Omicron BA.1 infected monkeys were also used for comparison given the limited number of animals used in this exploratory NHP study.<br>Sample size in the NHP therapeutic study was sixteen female cynomolgus macaques (Mauritian-origin) aged 3–6 years that were randomly assigned between the control (n=4) and two treated groups (n=6 each). In an analogous manner to the hamster studies, the 1- to 2-log reduction in viral RNA detected in the trachea, nasopharyngeal and BAL samples is sufficient to observe statistically significant differences between treated and untreated NHPs using the number of animals.<br>Cryopreserved effector cell from five healthy donors collected prior to 2019 (pre-pandemic) were used for the antibody dependent cellular cytotoxicity assays.
Data exclusions	No data was excluded from the in vitro studies except in the rare case of technical failure. In hamster challenge studies, no control animals were excluded. In treated groups, animals with undetectable levels of serum antibodies were excluded from the analysis as this indicated a technical failure in the drug administration.
Replication	Experimental assay were performed in biological duplicates, triplicates or more as indicated in the figure legends. Each in vitro study was repeated in independent experiments on separate days giving consistent results with representative data displayed in figures.
Randomization	Hamsters in prophylactic protection study and monkeys in both prophylactic protection and therapeutic treatment studies were randomly allocated to the different control and mAb treatment arms.
Blinding	Blinding was performed whereby the technician analyzing the samples for RNA and virus titration were not aware of the treatment groups being evaluated.

Reporting for specific materials, systems and methods

We require information from authors about some types of materials, experimental systems and methods used in many studies. Here, indicate whether each material, system or method listed is relevant to your study. If you are not sure if a list item applies to your research, read the appropriate section before selecting a response.

Materials & experimental systems

n/a	Involved in the study
☐	☒ Antibodies
☐	☒ Eukaryotic cell lines
☒	☐ Palaeontology and archaeology
☐	☒ Animals and other organisms
☐	☒ Human research participants
☒	☐ Clinical data
☒	☐ Dual use research of concern

Methods

n/a	Involved in the study
☒	☐ ChIP-seq
☐	☒ Flow cytometry
☒	☐ MRI-based neuroimaging

Antibodies

Antibodies used

Human mAbs were produced and purified at the EPFL protein production and structure core facility. These include IgG1 antibodies P2G3 LS, P5C3 LS, AZD8895, AZD1061, S309, REGN10933, REGN10987 and ADG-2. A clinical lot of Sotrovimab from Vir/GSK was used in the live virus cytopatic effect assay and pseudoviral assays. S309 recombinantly produced antibody was used for the competitive binding studies.
Mouse anti-human CD19 APC-Cy7 (BD Biosciences; Cat#557791, Clone SJ25C1; Lot 0164389; 5 µl titration), mouse anti-human CD3-BV510 (BD Biosciences; Cat#563109; Clone UCHT1; Lot 9137669;1 µl titration), mouse anti-human IgM-FITC (Biolegend; Cat#314506, clone MHM-88; Lot B268793; 2 µl titration), mouse anti-human IgD PE-CF594 (BD Biosciences; Cat#562540; Clone IA6-2; Lot 1097701; 3 µl titration), mouse anti-human CD27-APC (BD Biosciences; Cat#558664; Clone: M-T271; Lot 0021050; 5 µl titration), mouse anti-human CD38-V450 (BD Biosciences; Cat#646851, Clone HB7; Lot 1239359; 5 µl titration) mAbs were used for antigen specific B cell sorting .
Mouse anti-human CD56-AF488 (BD Biosciences; Cat#557699; Clone B159; Lot 1060238; 5 µl titration), mouse anti-human CD16-FITC (BD Biosciences; Cat#555406, Clone 3G8; Lot 3351672; 2 µl titration), mouse anti-human CD4-PECF594 (BD Biosciences; Cat#5562316, Clone RPA-T4; Lot7194657; 2 µl titration) and Annexin V- APC (Invitrogen, Cat#88-8102-72; Lot0282663; 2 µl titration) were used for ADCC validation tests and anti-HLA class I antibody as positive control (Invivogen; Cat #MA1-19027, Clone W6/32; 5 ng/ml)
anti-mouse IgG-PE secondary antibody (OneLambda Thermo Fisher; Cat# P-21129; Lot 2430385; 1 in 100 dilution) was used in the Spike-ACE2 Luminex assay and anti-human IgG-PE secondary antibody (OneLambda Thermo Fisher; Cat # H10104; Lot C10; 1 in 100 dilution) was used for the Spike Luminex binding assay

Validation

All anti-SARS-CoV-2 antibodies were validated in: 1) binding studies using Spike proteins, 2) lentiviral neutralization assays pseudotyped with Spike proteins from different SARS-CoV-2 variants and 3) live virus cytopathic effect neutralization assays. All values obtained for benchmark antibodies were consistent with published results. Secondary antibodies and flow cytometry antibodies were validated by vendors and in-house with positive and negative controls performed in parallel with each study. Vendor validation studies includes testing side by side with prior batches as reference so that the new batch provides consistent performance in the intended application. Comparison of fluorochrome-conjugated efficiency for different lots of antibody during manufacturing and use, evaluation of antigen positive cell populations in a continuum from low to high levels of target to ensure consistent sensitivity and specificity.

Eukaryotic cell lines

Policy information about cell lines

Cell line source(s)

ExpiCHO cells were obtained from ThermoFischer, HEK 293T, B95-8, U937, Vero E6 and Calu-3 cells from ATCC, 293T-ACE2 cells were generated in house through infection with a lentivirus to integrate the human ACE2 gene into the 293T genome as described in Fenwick et al, Cell Reports, 2021. Cell sorting was used to select for the pooled population of 293T cells that expressed high levels of cell surface ACE2. 3T3ms CD40L and CEM-NKR-CCR5-Luc cells were obtained from the NIH AIDS Reagent program. CEM-NKR-CCR5-Luc cells were used to generate stables expressing the original Wuhan 2019-nCoV were generated using a lentiviral system in the same manner as the human ACE2 stables.

Authentication

All cells grew as expected and stable cell lines were verified by flow cytometry to ensure cell surface expression of either human ACE2 on 293T cells or 2019-nCoV Spike on CEM-NKR-Luc cells.

Mycoplasma contamination

Cell lines were tested and found to be negative for mycoplasma.

Commonly misidentified lines
(See ICLAC register)

There were no commonly misidentified cell lines used in these studies.

Animals and other organisms

Policy information about studies involving animals; ARRIVE guidelines recommended for reporting animal research

Laboratory animals

Wild-type Syrian Golden hamsters (Mesocricetus auratus) were purchased from Janvier Laboratories and were housed in ventilated isolator cages (IsoCage N Biocontainment System, Tecniplast) with ad libitum access to food and water and cage enrichment (wood block). The animals (female hamsters of 6-8 weeks age) were acclimated for 4 days prior to study start. Housing conditions and experimental procedures were approved by the ethics committee of animal experimentation of KU Leuven.

Cynomolgus macaques (Macaca fascicularis) were female aged 3-6 years and originating from Mauritian AAALAC certified breeding centers were used in this study. All animals were housed within IDMIT animal facilities at CEA, Fontenay-aux-Roses under BSL-2 and BSL-3 containment when necessary (Animal facility authorization #D92-032-02, Préfecture des Hauts de Seine, France) and in compliance with European Directive 2010/63/EU, the French regulations and the Standards for Human Care and Use of Laboratory Animals, of the Office for Laboratory Animal Welfare (OLAW, assurance number #A5826-01, US). Animals tested negative for Campylobacter, Yersinia, Shigella and Salmonella before being use in the study.
The protocols were approved by the institutional ethical committee "Comité d'Ethique en Expérimentation Animale du Commissariat à l'Energie Atomique et aux Energies Alternatives" (CEtEA #44) under statement number A20-011. The study was authorized by the "Research, Innovation and Education Ministry" under registration number APAFIS#24434-2020030216532863. All information on the ethics committee is available at https://cache.media.enseignementsup-recherche.gouv.fr/file/utilisation_des_animaux_fins_scientifiques/22/1/comiteethiqueea17_juin2013_257221.pdf.

Wild animals: No wild animals were used in this study.

Field-collected samples: No field-collected samples were used in this study.

Ethics oversight: Live virus-related work was conducted in the high-containment A3 and BSL3+ facilities of the KU Leuven Rega Institute (3CAPS) under licenses AMV 30112018 SBB 219 2018 0892 and AMV 23102017 SBB 219 20170589 according to institutional guidelines. Experimental procedures were approved by the ethics committee of animal experimentation of KU Leuven.
In NHP studies, animals were housed within IDMIT animal facilities at CEA, Fontenay-aux-Roses under BSL-2 and BSL-3 containment when necessary (Animal facility authorization #D92-032-02, Préfecture des Hauts de Seine, France) and in compliance with European Directive 2010/63/EU, the French regulations and the Standards for Human Care and Use of Laboratory Animals, of the Office for Laboratory Animal Welfare (OLAW, assurance number #A5826-01, US). Animals tested negative for Campylobacter, Yersinia, Shigella and Salmonella before being use in the study.

Note that full information on the approval of the study protocol must also be provided in the manuscript.

Human research participants

Policy information about studies involving human research participants

Population characteristics: Participants in the ImmunoCov and ImmunoVax studies were recruited by the Immunology and Allergy Service, Lausanne University Hospital and consisted of COVID-19 patients (ImmunoCov), vaccinated donors that are immune compromised (ImmunoVax) or vaccinated healthy control donors (ImmunoVax). All donors were adults of varying age and no restriction on gender.

Recruitment: Donors were recruited through the Lausanne University Hospital as patients or as healthy volunteers selected to have a similar age distribution to the patient population. This recruitment strategy would not influence the nature of our studies.

Ethics oversight: Study design and use of subject samples were approved by the Institutional Review Board of the Lausanne University Hospital and the 'Commission d'éthique du Canton de Vaud' (CER-VD with trial reference numbers 2020-00620 and 2021-00041, respectively).

Note that full information on the approval of the study protocol must also be provided in the manuscript.

Flow Cytometry

Plots

Confirm that:

- [x] The axis labels state the marker and fluorochrome used (e.g. CD4-FITC).
- [x] The axis scales are clearly visible. Include numbers along axes only for bottom left plot of group (a 'group' is an analysis of identical markers).
- [x] All plots are contour plots with outliers or pseudocolor plots.
- [x] A numerical value for number of cells or percentage (with statistics) is provided.

Methodology

Sample preparation: Cell lines grown in suspension were stained directly for analysis.

Instrument: FACSAria and LSR II instruments from BD

Software: FlowJo version 10.7.1 and Cytobank softwares

Cell population abundance: Not applicable for most studies since homogeneous cell lines were used. In antigen specific B cell sorting, Spike positive B cells represented approximately 1-3% of memory B cells.

Gating strategy: Cell gating examples will be provided in an updated supplemental information section.
In the cell sorting of SARS-CoV-2 Spike beta variant specific B cells, gates applied were SSC-A vs. FSC-A to remove cell debris and gate on B cells then FSC-H vs FSC-A to select single cells. Viable B cells were then identified as being CD19 positive, CD3 negative and Aqua negative. Memory B cells were selected at CD27 positive and IgM negative and finally antigen specific

memory B cells were sorted based on staining with biotinylated Spike trimer bound to streptavidin-PE tetramers.
In ADCP assay performed with U937 cells, gates applied were SSC-A vs. FSC-A to remove cell debris and gate on U937 cells then FSC-H vs FSC-A to select single cells. Phagocytosis of Spike coated fluorescent beads was monitored within the Aqua negative, live U937 cell population.

☒ Tick this box to confirm that a figure exemplifying the gating strategy is provided in the Supplementary Information.
